# MEME-LaB: motif analysis in clusters

**DOI:** 10.1093/bioinformatics/btt248

**Published:** 2013-05-14

**Authors:** Paul Brown, Laura Baxter, Richard Hickman, Jim Beynon, Jonathan D. Moore, Sascha Ott

**Affiliations:** ^1^Warwick Systems Biology Centre and ^2^School of Life Sciences, University of Warwick, Coventry, CV4 7AL, UK

## Abstract

**Summary:** Genome-wide expression analysis can result in large numbers of clusters of co-expressed genes. Although there are tools for *ab initio* discovery of transcription factor-binding sites, most do not provide a quick and easy way to study large numbers of clusters. To address this, we introduce a web tool called MEME-LaB. The tool wraps MEME (an *ab initio* motif finder), providing an interface for users to input multiple gene clusters, retrieve promoter sequences, run motif finding and then easily browse and condense the results, facilitating better interpretation of the results from large-scale datasets.

**Availability:** MEME-LaB is freely accessible at: http://wsbc.warwick.ac.uk/wsbcToolsWebpage/.

**Contact:** p.e.brown@warwick.ac.uk

**Supplementary information:** Supplementary data are available at *Bioinformatics* online.

## 1 INTRODUCTION

Analyzing microarray expression data using cluster analysis is a common and frequently performed task in functional genomics. Typically, a large number of clusters are produced, each containing a large number of genes (e.g. 50 clusters of 200 genes). Each cluster is predicted to contain a set of genes that are co-expressed and as such would be expected to share common regulatory features, such as transcription factor-binding sites (TFBSs). There are established methods and tools for predicting known TFBSs (such as Athena, O’Connor *et al.*, 2005), but *ab initio* motif discovery remains an important aspect to consider. Several tools exist to perform this task, and MEME in particular is a well-recognized suite for motif discovery (Bailey *et al.*, 2006), but the MEME web suite offers limited usage on large numbers of clusters and subsequent navigation and post-processing of the results. Machanick and Bailey (2012) provide a web tool (MEME-ChIP) that is specifically tailored towards ChIP-seq data, providing a useful expansion in MEME’s functionality.

Here, we describe a web tool called MEME-LaB (MEME Launcher and Browser), which wraps the MEME tool in ways that are ideally suited to the task of *ab initio* motif finding in co-expressed gene clusters: (i) users can input multiple gene clusters at once. (ii) Promoter sequences are automatically retrieved from a local database or from a user-specified file. (iii) MEME is run on all clusters simultaneously, and the results are presented in a condensed and navigable format. (iv) Identified motifs are compared for similarity with known TFBS motifs.

## 2 IMPLEMENTATION

### 2.1 General workflow

The MEME-LaB web service is designed to simplify the task of identifying putative TFBSs in the promoters of co-expressed gene clusters, and it provides an easy way to navigate through and filter the results.

### 2.2 Input

Users are not required to register for the service and are automatically logged in as a guest user. Gene clusters are uploaded as a simple tab-separated file consisting of two columns: the first for numbers identifying the clusters, and the second for gene IDs. For the *Arabidopsis* genome, sequences will be automatically retrieved from a local database; for all other genomes, a second file containing promoter sequences in FASTA format is uploaded by the user.

### 2.3 Processing

Uploaded files are verified for validity, and the user warned of any detected errors in the input. Users specify the minimum and maximum length of promoter regions to search, between 50 and 1000 nt in length, and optionally to stop at a neighbouring gene if there is one present in this region. Users select the number of motifs to find per cluster, within a minimum and maximum motif length (6–20 nt). Details of comparisons with known motifs from JASPAR (Bryne *et al.*, 2008) and PLACE (Higo *et al.*, 1999) are provided in the output (users with a valid login for TRANSFAC can also compare motifs with TRANSFAC motifs on our server). An email is sent to the address provided notifying when the job is complete and results ready for retrieval.

### 2.4 Output

Results are provided as interactive html pages, and they can also be downloaded. For each cluster, the specified number of motifs is identified using the MEME algorithm and displayed as motif logos. Additional information for each motif is displayed, including its distribution among the input set, positional bias, strand bias and similarity to known motifs.

## 3 EXAMPLE RESULTS

**Fig. 1.**
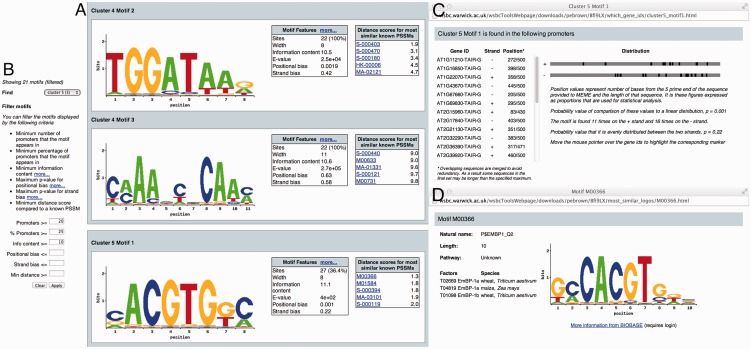
Screenshot of a typical MEME-LaB result. Motifs are displayed as motif logos, and information about each motif is shown (**A**). Results can be filtered on motif properties (**B**). Additional information on positional distribution (**C**) and properties of similar motifs (**D**) are accessed in pop-up windows

We demonstrate the functionality offered by MEME-LaB, using co-expression clusters derived from a time-course microarray experiment of *Arabidopsis* responses to infection with *Botrytis cinerea* (Windram *et al.*, 2012) (input files and the complete results are available as Supplementary Data S1). The usefulness of the tool is demonstrated by being able to easily browse motifs for all clusters on a single webpage and to reduce a large set of motifs to the most informative results based on motif properties (Fig. 1A and B). Additional information on each motif’s positional distribution among the input set is provided (Fig. 1C). For each *ab initio* motif predicted, up to five of the most similar known motifs are listed, with a distance measure, and additional information and links are provided (Fig. 1A and D). In the example result view (Fig. 1), filtering to show only motifs that occur in 25% or more of sequences in a cluster, occurring in ≥20 sites and have an information content >10 has resulted in 21 of the possible 220 motifs being displayed. MEME identified motifs similar to I-box (Fig. 1A, top) and G-box (Fig. 1A, bottom), which are consistent with previous findings, but also a third motif (Fig. 1A, middle) that is not closely similar to any known motif but is present in all the sequences in cluster 4. The MEME-LaB service makes existing functionality more widely and more easily applicable, enabling the identification of significant motifs in large co-expression cluster datasets.

*Funding*: Biotechnology and Biological Sciences Research Council (BBSRC) (BB/F005806/1 to P.B., L.B., J.D.M., J.B. and S.O.); Engineering and Physical Sciences Research Council/BBSRC–funded Warwick Systems Biology Doctoral Training Centre (to R.H.).

*Conflict of Interest*: none declared.

## Supplementary Material

Supplementary Data
