# Supplementary material for: MEME-LaB: motif analysis in clusters
Source: Bioinformatics. 2013 May 14;29(13):1696–7. doi: 10.1093/bioinformatics/btt248 (PMC3694638; doi:10.1093/bioinformatics/btt248)
Supplement: Supplementary Data [file supp_29_13_1696__index.html]

MEME-LaB: motif analysis in clusters — Supplementary Data 

# MEME-LaB: motif analysis in clusters

## 

files

**Files in this Data Supplement:**

- Supplementary Data - zip file
