# Supplementary material for: MEME-LaB: motif analysis in clusters
Source: Bioinformatics. 2013 May 14;29(13):1696–7. doi: 10.1093/bioinformatics/btt248 (PMC3694638; doi:10.1093/bioinformatics/btt248)
Supplement: Supplementary Data [file supp_btt248_Supplementary_Data.zip › Supplementary_Data/Input_Files/Input_Readme.rtf]

These files are provided as example input files to the MEME-LaB tool.44botrytisclusters.txt is a file containing gene ids.TAIR10_upstream_500_20101028.fasta is a fasta file of promoter sequences.
