# Supplementary figures and images for: MEME-LaB: motif analysis in clusters (part 2 of 2)
Source: Bioinformatics. 2013 May 14;29(13):1696–7. doi: 10.1093/bioinformatics/btt248 (PMC3694638; doi:10.1093/bioinformatics/btt248)

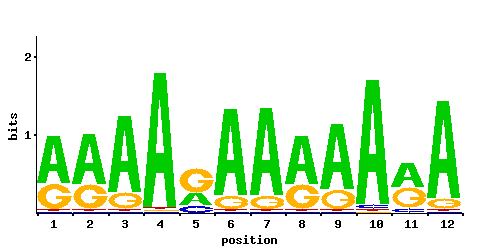

Supplement: Supplementary Data [file supp_btt248_Supplementary_Data.zip › Supplementary_Data/Results_Files/logos_non-repeatmasked_500bp/29/29-1.png]

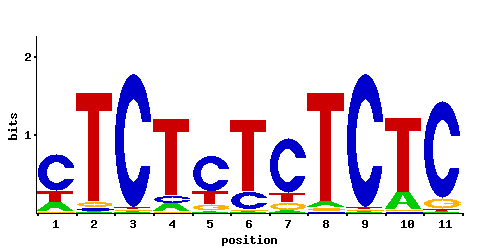

Supplement: Supplementary Data [file supp_btt248_Supplementary_Data.zip › Supplementary_Data/Results_Files/logos_non-repeatmasked_500bp/29/29-2.png]

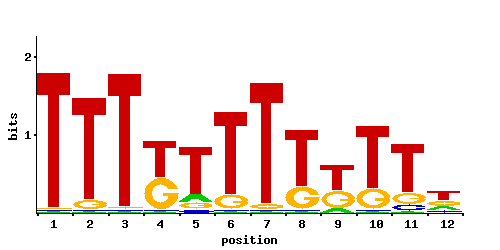

Supplement: Supplementary Data [file supp_btt248_Supplementary_Data.zip › Supplementary_Data/Results_Files/logos_non-repeatmasked_500bp/29/29-3.png]

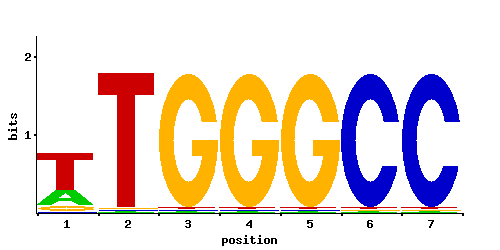

Supplement: Supplementary Data [file supp_btt248_Supplementary_Data.zip › Supplementary_Data/Results_Files/logos_non-repeatmasked_500bp/29/29-4.png]

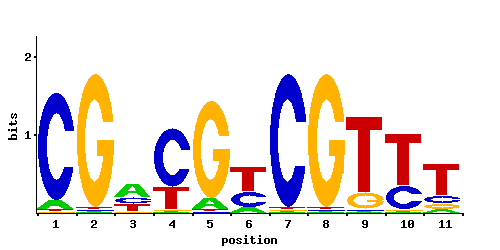

Supplement: Supplementary Data [file supp_btt248_Supplementary_Data.zip › Supplementary_Data/Results_Files/logos_non-repeatmasked_500bp/29/29-5.png]

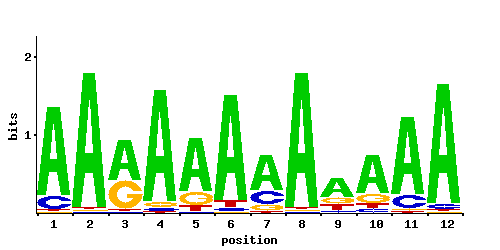

Supplement: Supplementary Data [file supp_btt248_Supplementary_Data.zip › Supplementary_Data/Results_Files/logos_non-repeatmasked_500bp/3/3-1.png]

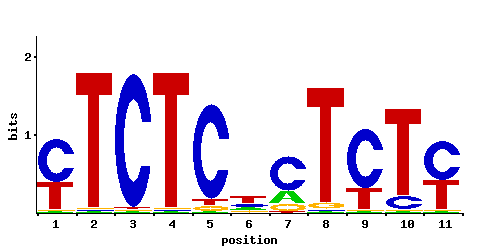

Supplement: Supplementary Data [file supp_btt248_Supplementary_Data.zip › Supplementary_Data/Results_Files/logos_non-repeatmasked_500bp/3/3-2.png]

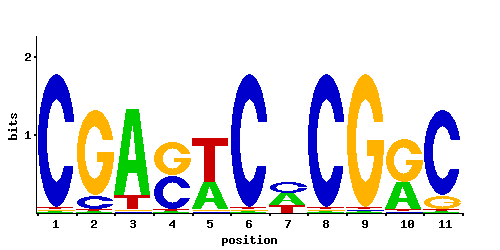

Supplement: Supplementary Data [file supp_btt248_Supplementary_Data.zip › Supplementary_Data/Results_Files/logos_non-repeatmasked_500bp/3/3-3.png]

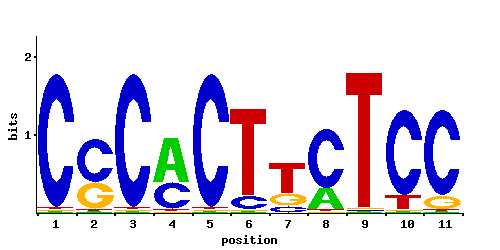

Supplement: Supplementary Data [file supp_btt248_Supplementary_Data.zip › Supplementary_Data/Results_Files/logos_non-repeatmasked_500bp/3/3-4.png]

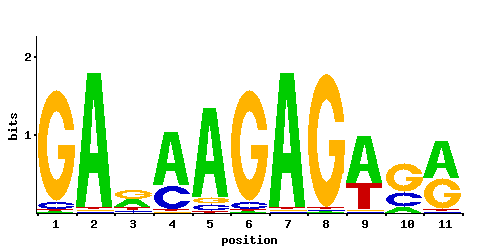

Supplement: Supplementary Data [file supp_btt248_Supplementary_Data.zip › Supplementary_Data/Results_Files/logos_non-repeatmasked_500bp/3/3-5.png]

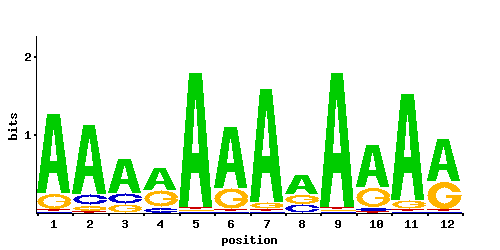

Supplement: Supplementary Data [file supp_btt248_Supplementary_Data.zip › Supplementary_Data/Results_Files/logos_non-repeatmasked_500bp/30/30-1.png]

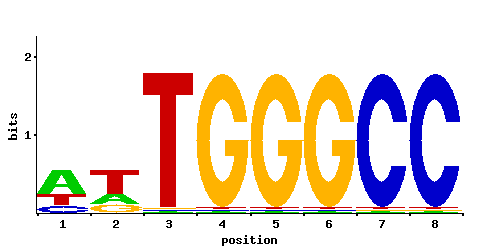

Supplement: Supplementary Data [file supp_btt248_Supplementary_Data.zip › Supplementary_Data/Results_Files/logos_non-repeatmasked_500bp/30/30-2.png]

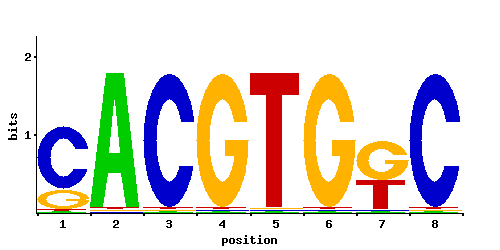

Supplement: Supplementary Data [file supp_btt248_Supplementary_Data.zip › Supplementary_Data/Results_Files/logos_non-repeatmasked_500bp/30/30-3.png]

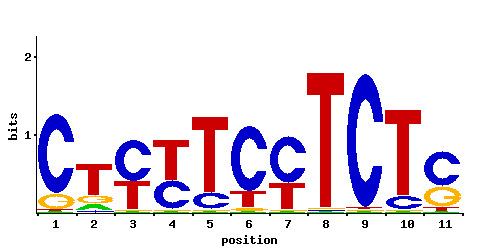

Supplement: Supplementary Data [file supp_btt248_Supplementary_Data.zip › Supplementary_Data/Results_Files/logos_non-repeatmasked_500bp/30/30-4.png]

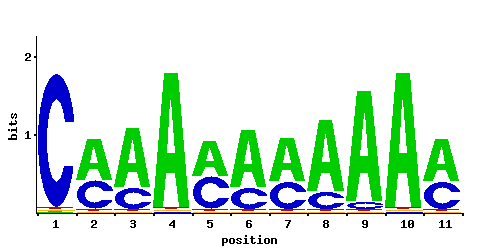

Supplement: Supplementary Data [file supp_btt248_Supplementary_Data.zip › Supplementary_Data/Results_Files/logos_non-repeatmasked_500bp/30/30-5.png]

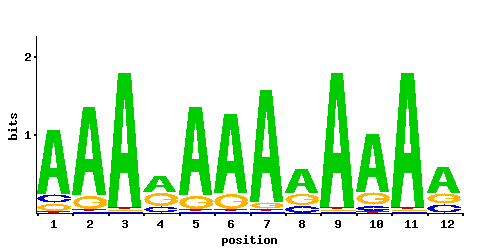

Supplement: Supplementary Data [file supp_btt248_Supplementary_Data.zip › Supplementary_Data/Results_Files/logos_non-repeatmasked_500bp/31/31-1.png]

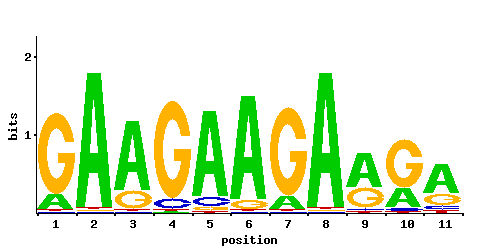

Supplement: Supplementary Data [file supp_btt248_Supplementary_Data.zip › Supplementary_Data/Results_Files/logos_non-repeatmasked_500bp/31/31-2.png]

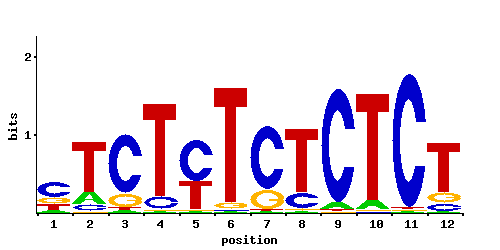

Supplement: Supplementary Data [file supp_btt248_Supplementary_Data.zip › Supplementary_Data/Results_Files/logos_non-repeatmasked_500bp/31/31-3.png]

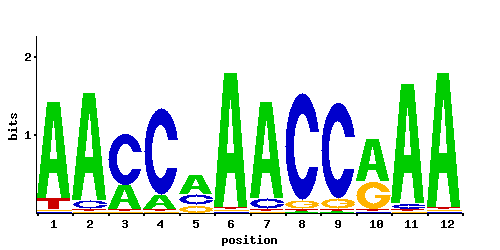

Supplement: Supplementary Data [file supp_btt248_Supplementary_Data.zip › Supplementary_Data/Results_Files/logos_non-repeatmasked_500bp/31/31-4.png]

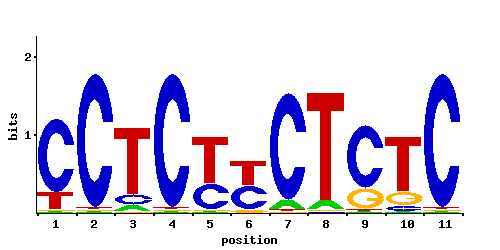

Supplement: Supplementary Data [file supp_btt248_Supplementary_Data.zip › Supplementary_Data/Results_Files/logos_non-repeatmasked_500bp/31/31-5.png]

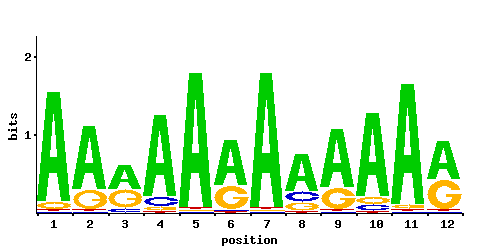

Supplement: Supplementary Data [file supp_btt248_Supplementary_Data.zip › Supplementary_Data/Results_Files/logos_non-repeatmasked_500bp/32/32-1.png]

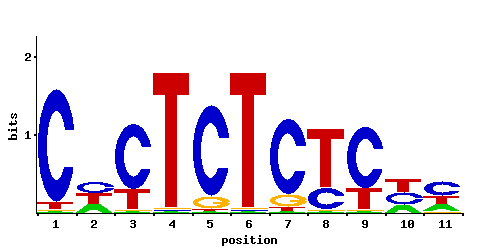

Supplement: Supplementary Data [file supp_btt248_Supplementary_Data.zip › Supplementary_Data/Results_Files/logos_non-repeatmasked_500bp/32/32-2.png]

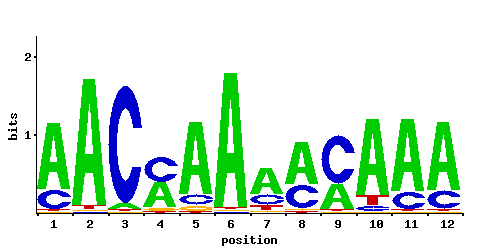

Supplement: Supplementary Data [file supp_btt248_Supplementary_Data.zip › Supplementary_Data/Results_Files/logos_non-repeatmasked_500bp/32/32-3.png]

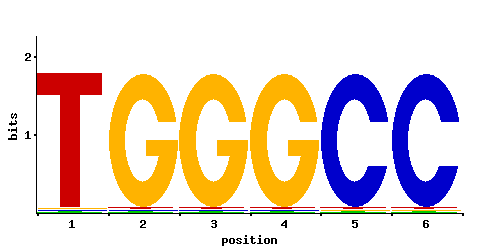

Supplement: Supplementary Data [file supp_btt248_Supplementary_Data.zip › Supplementary_Data/Results_Files/logos_non-repeatmasked_500bp/32/32-4.png]

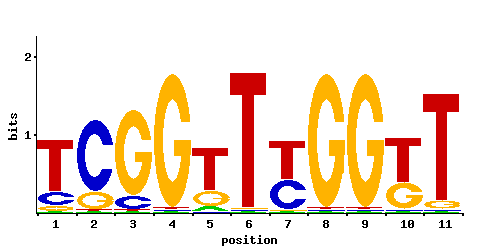

Supplement: Supplementary Data [file supp_btt248_Supplementary_Data.zip › Supplementary_Data/Results_Files/logos_non-repeatmasked_500bp/32/32-5.png]

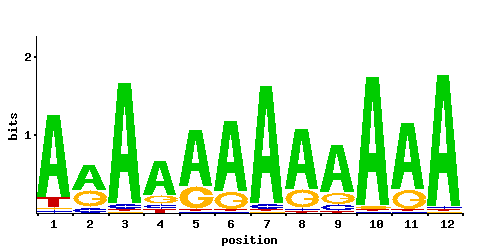

Supplement: Supplementary Data [file supp_btt248_Supplementary_Data.zip › Supplementary_Data/Results_Files/logos_non-repeatmasked_500bp/33/33-1.png]

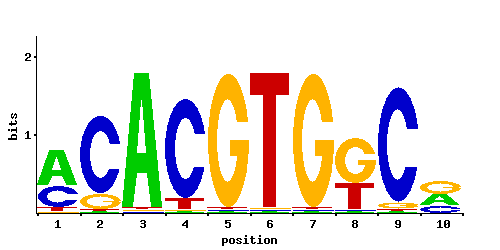

Supplement: Supplementary Data [file supp_btt248_Supplementary_Data.zip › Supplementary_Data/Results_Files/logos_non-repeatmasked_500bp/33/33-2.png]

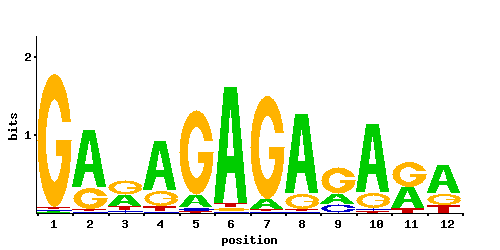

Supplement: Supplementary Data [file supp_btt248_Supplementary_Data.zip › Supplementary_Data/Results_Files/logos_non-repeatmasked_500bp/33/33-3.png]

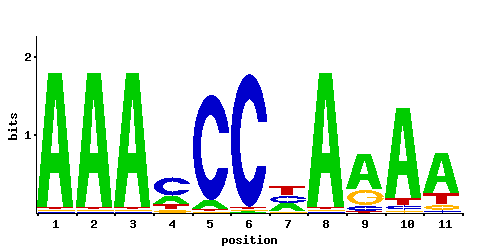

Supplement: Supplementary Data [file supp_btt248_Supplementary_Data.zip › Supplementary_Data/Results_Files/logos_non-repeatmasked_500bp/33/33-4.png]

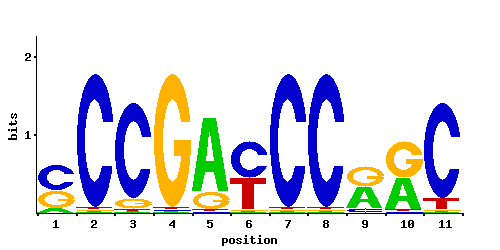

Supplement: Supplementary Data [file supp_btt248_Supplementary_Data.zip › Supplementary_Data/Results_Files/logos_non-repeatmasked_500bp/33/33-5.png]

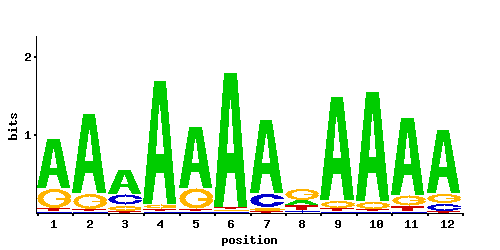

Supplement: Supplementary Data [file supp_btt248_Supplementary_Data.zip › Supplementary_Data/Results_Files/logos_non-repeatmasked_500bp/34/34-1.png]

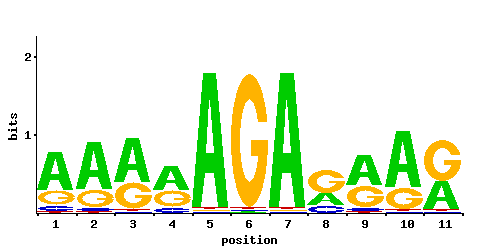

Supplement: Supplementary Data [file supp_btt248_Supplementary_Data.zip › Supplementary_Data/Results_Files/logos_non-repeatmasked_500bp/34/34-2.png]

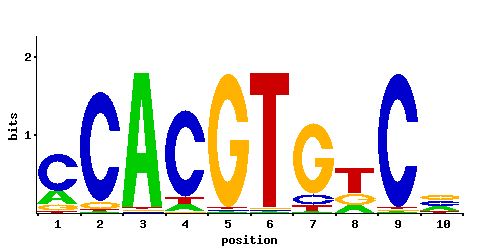

Supplement: Supplementary Data [file supp_btt248_Supplementary_Data.zip › Supplementary_Data/Results_Files/logos_non-repeatmasked_500bp/34/34-3.png]

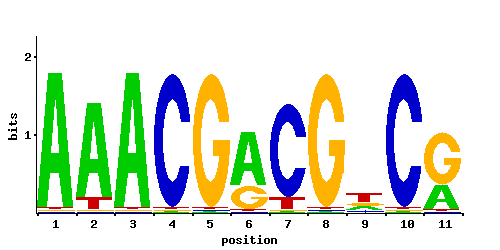

Supplement: Supplementary Data [file supp_btt248_Supplementary_Data.zip › Supplementary_Data/Results_Files/logos_non-repeatmasked_500bp/34/34-4.png]

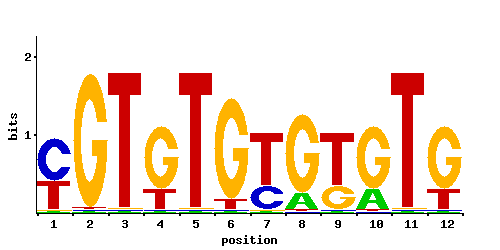

Supplement: Supplementary Data [file supp_btt248_Supplementary_Data.zip › Supplementary_Data/Results_Files/logos_non-repeatmasked_500bp/34/34-5.png]

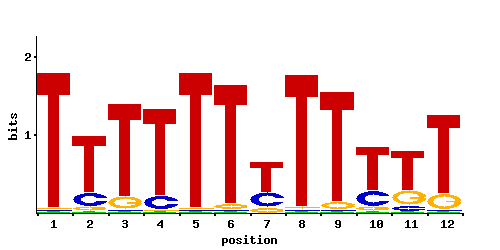

Supplement: Supplementary Data [file supp_btt248_Supplementary_Data.zip › Supplementary_Data/Results_Files/logos_non-repeatmasked_500bp/36/36-1.png]

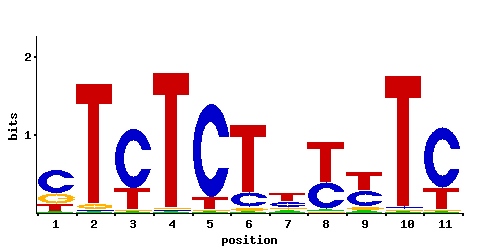

Supplement: Supplementary Data [file supp_btt248_Supplementary_Data.zip › Supplementary_Data/Results_Files/logos_non-repeatmasked_500bp/36/36-2.png]

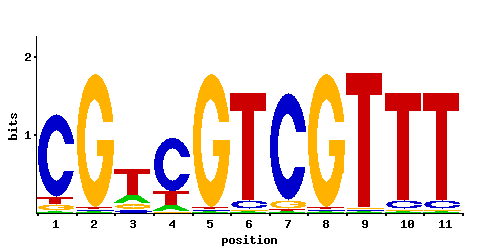

Supplement: Supplementary Data [file supp_btt248_Supplementary_Data.zip › Supplementary_Data/Results_Files/logos_non-repeatmasked_500bp/36/36-3.png]

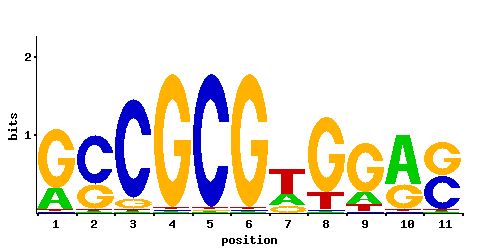

Supplement: Supplementary Data [file supp_btt248_Supplementary_Data.zip › Supplementary_Data/Results_Files/logos_non-repeatmasked_500bp/36/36-4.png]

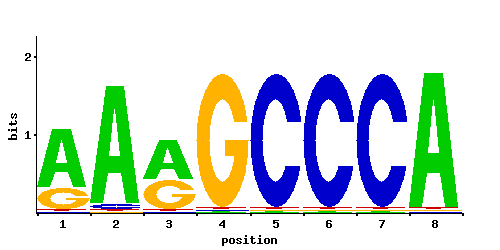

Supplement: Supplementary Data [file supp_btt248_Supplementary_Data.zip › Supplementary_Data/Results_Files/logos_non-repeatmasked_500bp/36/36-5.png]

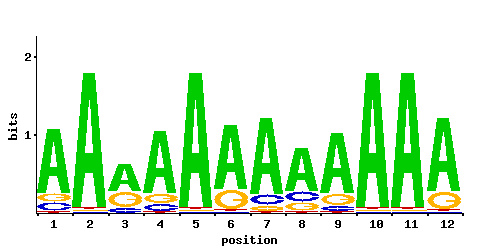

Supplement: Supplementary Data [file supp_btt248_Supplementary_Data.zip › Supplementary_Data/Results_Files/logos_non-repeatmasked_500bp/37/37-1.png]

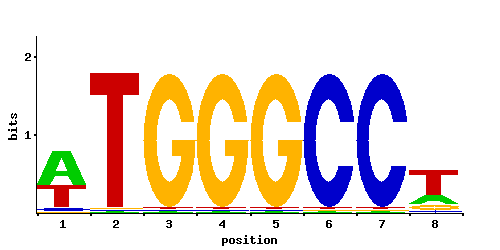

Supplement: Supplementary Data [file supp_btt248_Supplementary_Data.zip › Supplementary_Data/Results_Files/logos_non-repeatmasked_500bp/37/37-2.png]

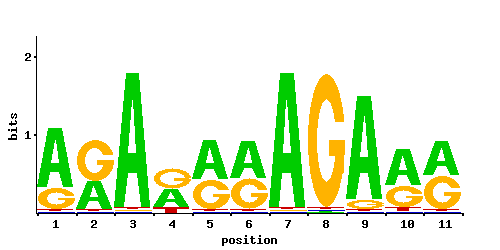

Supplement: Supplementary Data [file supp_btt248_Supplementary_Data.zip › Supplementary_Data/Results_Files/logos_non-repeatmasked_500bp/37/37-3.png]

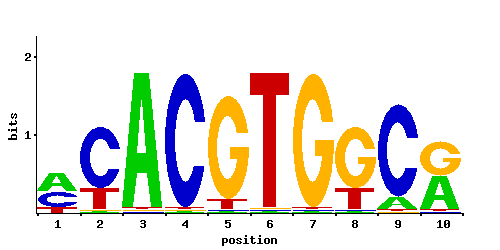

Supplement: Supplementary Data [file supp_btt248_Supplementary_Data.zip › Supplementary_Data/Results_Files/logos_non-repeatmasked_500bp/37/37-4.png]

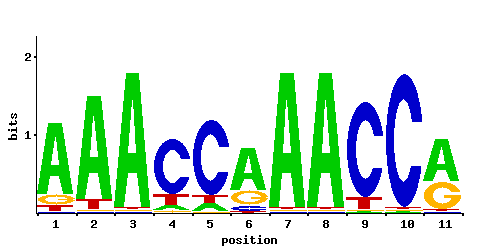

Supplement: Supplementary Data [file supp_btt248_Supplementary_Data.zip › Supplementary_Data/Results_Files/logos_non-repeatmasked_500bp/37/37-5.png]

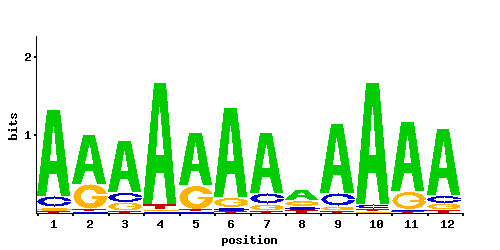

Supplement: Supplementary Data [file supp_btt248_Supplementary_Data.zip › Supplementary_Data/Results_Files/logos_non-repeatmasked_500bp/38/38-1.png]

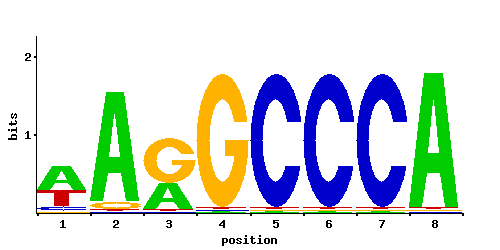

Supplement: Supplementary Data [file supp_btt248_Supplementary_Data.zip › Supplementary_Data/Results_Files/logos_non-repeatmasked_500bp/38/38-2.png]

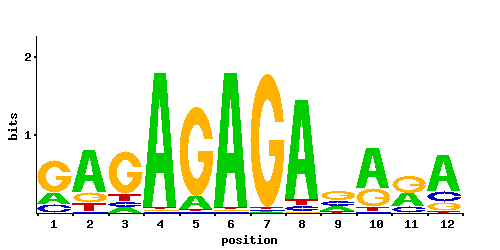

Supplement: Supplementary Data [file supp_btt248_Supplementary_Data.zip › Supplementary_Data/Results_Files/logos_non-repeatmasked_500bp/38/38-3.png]

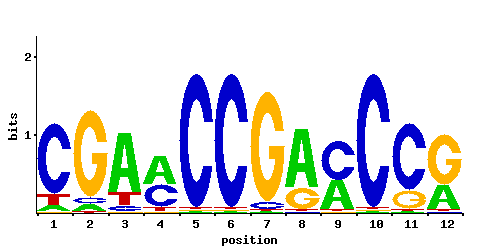

Supplement: Supplementary Data [file supp_btt248_Supplementary_Data.zip › Supplementary_Data/Results_Files/logos_non-repeatmasked_500bp/38/38-4.png]

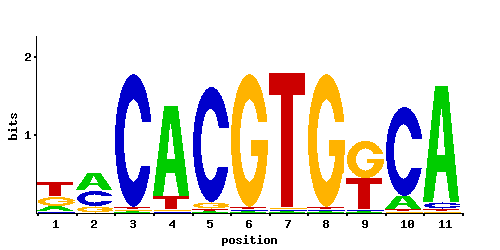

Supplement: Supplementary Data [file supp_btt248_Supplementary_Data.zip › Supplementary_Data/Results_Files/logos_non-repeatmasked_500bp/38/38-5.png]

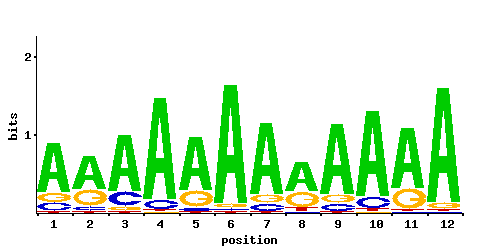

Supplement: Supplementary Data [file supp_btt248_Supplementary_Data.zip › Supplementary_Data/Results_Files/logos_non-repeatmasked_500bp/39/39-1.png]

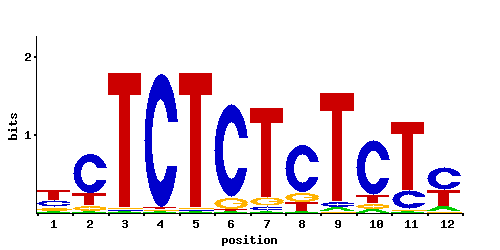

Supplement: Supplementary Data [file supp_btt248_Supplementary_Data.zip › Supplementary_Data/Results_Files/logos_non-repeatmasked_500bp/39/39-2.png]

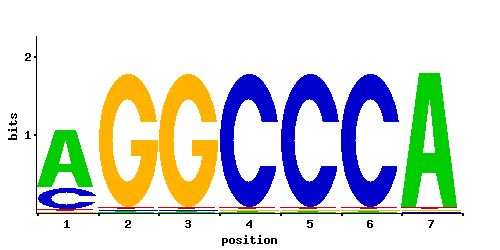

Supplement: Supplementary Data [file supp_btt248_Supplementary_Data.zip › Supplementary_Data/Results_Files/logos_non-repeatmasked_500bp/39/39-3.png]

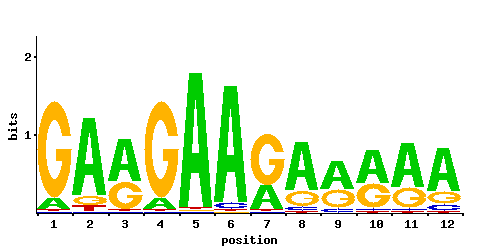

Supplement: Supplementary Data [file supp_btt248_Supplementary_Data.zip › Supplementary_Data/Results_Files/logos_non-repeatmasked_500bp/39/39-4.png]

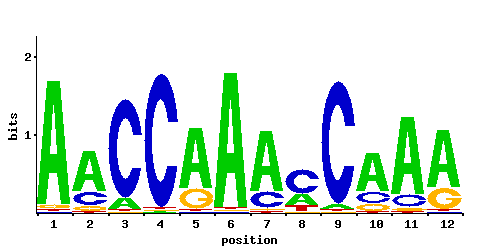

Supplement: Supplementary Data [file supp_btt248_Supplementary_Data.zip › Supplementary_Data/Results_Files/logos_non-repeatmasked_500bp/39/39-5.png]

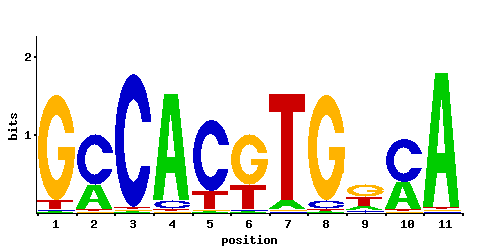

Supplement: Supplementary Data [file supp_btt248_Supplementary_Data.zip › Supplementary_Data/Results_Files/logos_non-repeatmasked_500bp/4/4-1.png]

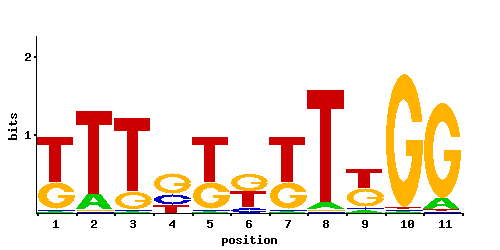

Supplement: Supplementary Data [file supp_btt248_Supplementary_Data.zip › Supplementary_Data/Results_Files/logos_non-repeatmasked_500bp/4/4-2.png]

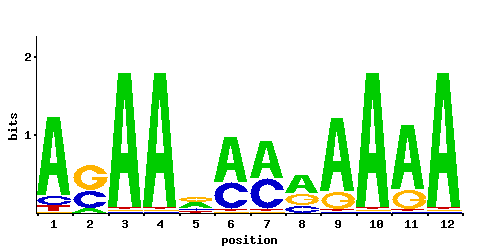

Supplement: Supplementary Data [file supp_btt248_Supplementary_Data.zip › Supplementary_Data/Results_Files/logos_non-repeatmasked_500bp/4/4-3.png]

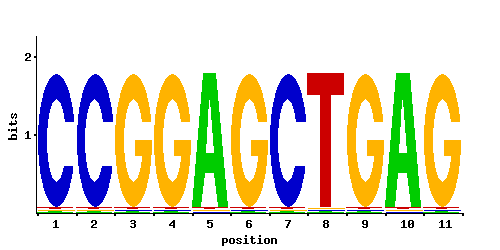

Supplement: Supplementary Data [file supp_btt248_Supplementary_Data.zip › Supplementary_Data/Results_Files/logos_non-repeatmasked_500bp/4/4-4.png]

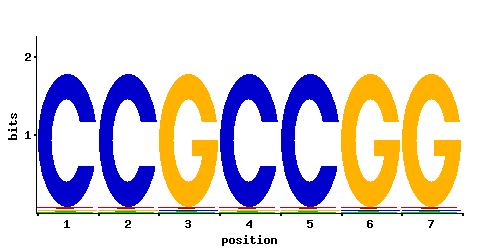

Supplement: Supplementary Data [file supp_btt248_Supplementary_Data.zip › Supplementary_Data/Results_Files/logos_non-repeatmasked_500bp/4/4-5.png]

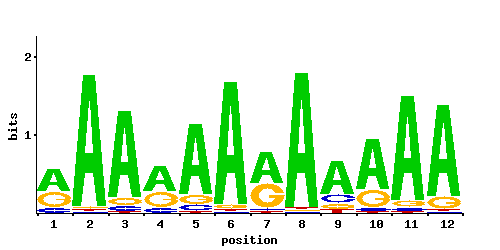

Supplement: Supplementary Data [file supp_btt248_Supplementary_Data.zip › Supplementary_Data/Results_Files/logos_non-repeatmasked_500bp/40/40-1.png]

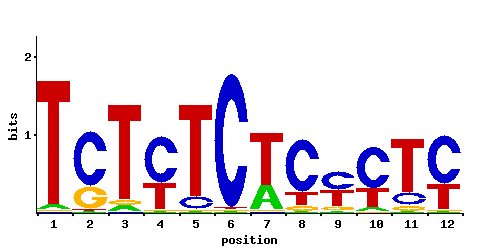

Supplement: Supplementary Data [file supp_btt248_Supplementary_Data.zip › Supplementary_Data/Results_Files/logos_non-repeatmasked_500bp/40/40-2.png]

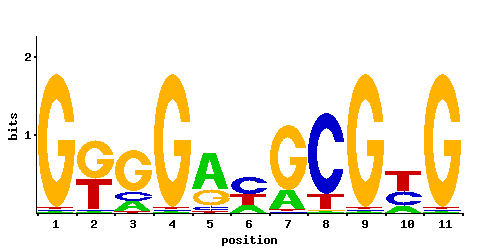

Supplement: Supplementary Data [file supp_btt248_Supplementary_Data.zip › Supplementary_Data/Results_Files/logos_non-repeatmasked_500bp/40/40-3.png]

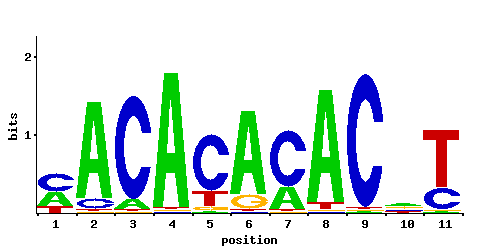

Supplement: Supplementary Data [file supp_btt248_Supplementary_Data.zip › Supplementary_Data/Results_Files/logos_non-repeatmasked_500bp/40/40-4.png]

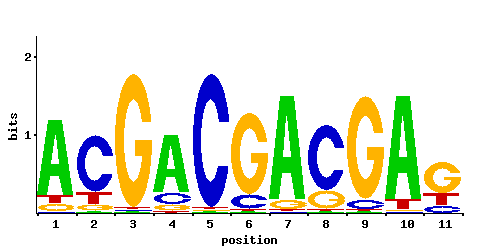

Supplement: Supplementary Data [file supp_btt248_Supplementary_Data.zip › Supplementary_Data/Results_Files/logos_non-repeatmasked_500bp/40/40-5.png]

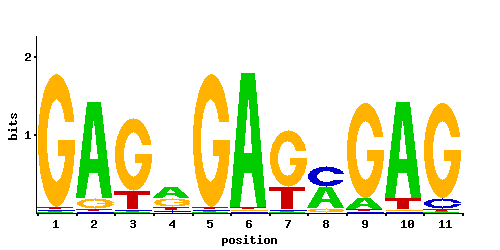

Supplement: Supplementary Data [file supp_btt248_Supplementary_Data.zip › Supplementary_Data/Results_Files/logos_non-repeatmasked_500bp/41/41-1.png]

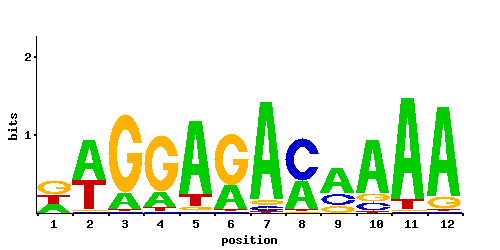

Supplement: Supplementary Data [file supp_btt248_Supplementary_Data.zip › Supplementary_Data/Results_Files/logos_non-repeatmasked_500bp/41/41-2.png]

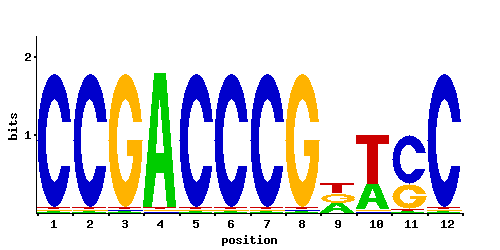

Supplement: Supplementary Data [file supp_btt248_Supplementary_Data.zip › Supplementary_Data/Results_Files/logos_non-repeatmasked_500bp/41/41-3.png]

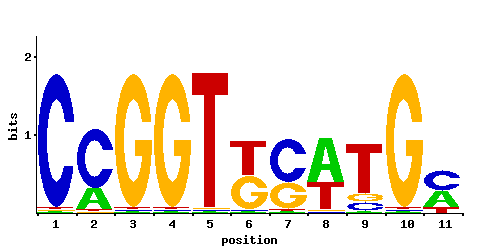

Supplement: Supplementary Data [file supp_btt248_Supplementary_Data.zip › Supplementary_Data/Results_Files/logos_non-repeatmasked_500bp/41/41-4.png]

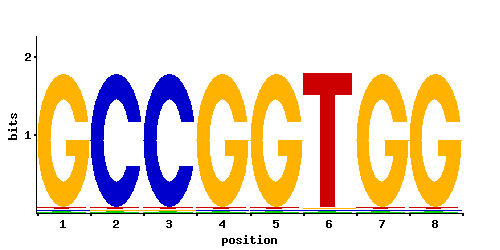

Supplement: Supplementary Data [file supp_btt248_Supplementary_Data.zip › Supplementary_Data/Results_Files/logos_non-repeatmasked_500bp/41/41-5.png]

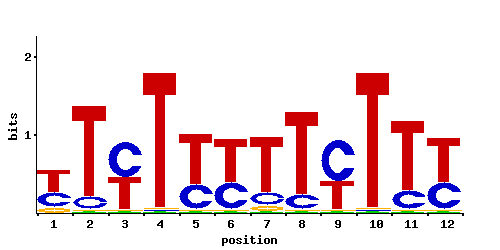

Supplement: Supplementary Data [file supp_btt248_Supplementary_Data.zip › Supplementary_Data/Results_Files/logos_non-repeatmasked_500bp/42/42-1.png]

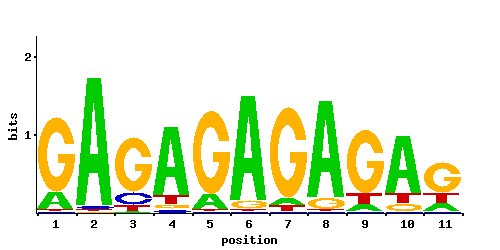

Supplement: Supplementary Data [file supp_btt248_Supplementary_Data.zip › Supplementary_Data/Results_Files/logos_non-repeatmasked_500bp/42/42-2.png]

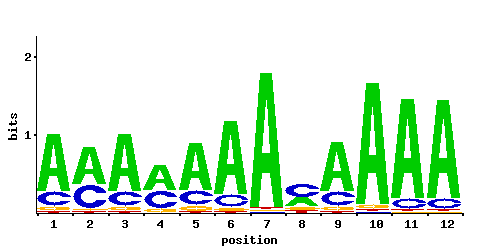

Supplement: Supplementary Data [file supp_btt248_Supplementary_Data.zip › Supplementary_Data/Results_Files/logos_non-repeatmasked_500bp/42/42-3.png]

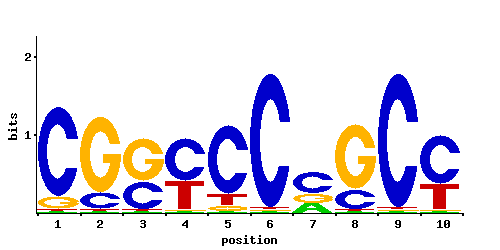

Supplement: Supplementary Data [file supp_btt248_Supplementary_Data.zip › Supplementary_Data/Results_Files/logos_non-repeatmasked_500bp/42/42-4.png]

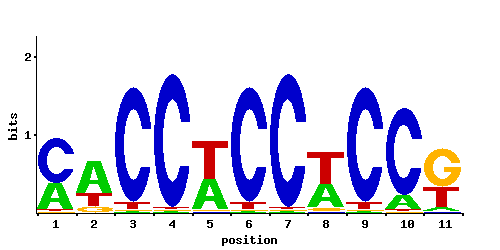

Supplement: Supplementary Data [file supp_btt248_Supplementary_Data.zip › Supplementary_Data/Results_Files/logos_non-repeatmasked_500bp/42/42-5.png]

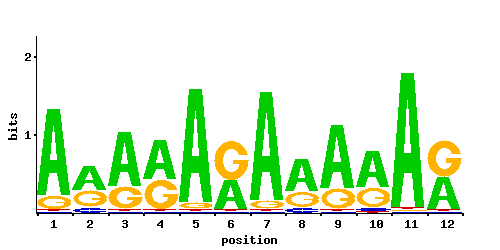

Supplement: Supplementary Data [file supp_btt248_Supplementary_Data.zip › Supplementary_Data/Results_Files/logos_non-repeatmasked_500bp/43/43-1.png]

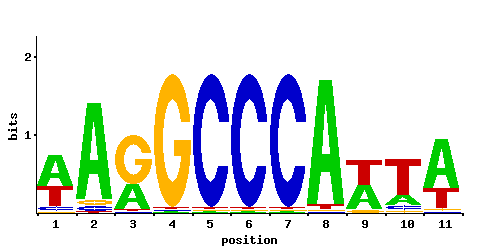

Supplement: Supplementary Data [file supp_btt248_Supplementary_Data.zip › Supplementary_Data/Results_Files/logos_non-repeatmasked_500bp/43/43-2.png]

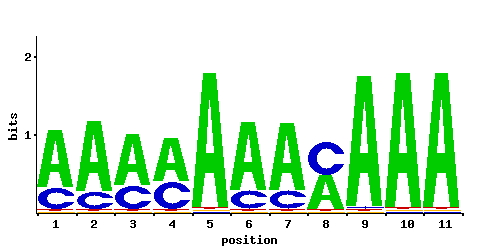

Supplement: Supplementary Data [file supp_btt248_Supplementary_Data.zip › Supplementary_Data/Results_Files/logos_non-repeatmasked_500bp/43/43-3.png]

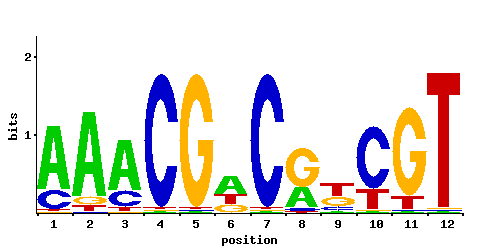

Supplement: Supplementary Data [file supp_btt248_Supplementary_Data.zip › Supplementary_Data/Results_Files/logos_non-repeatmasked_500bp/43/43-4.png]

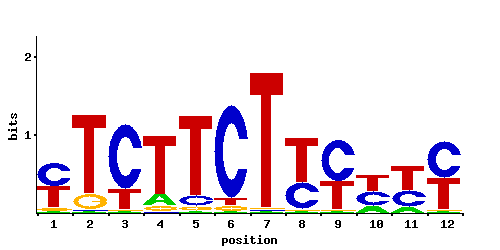

Supplement: Supplementary Data [file supp_btt248_Supplementary_Data.zip › Supplementary_Data/Results_Files/logos_non-repeatmasked_500bp/43/43-5.png]

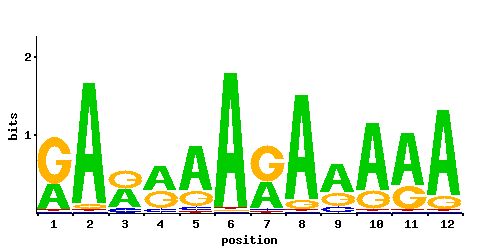

Supplement: Supplementary Data [file supp_btt248_Supplementary_Data.zip › Supplementary_Data/Results_Files/logos_non-repeatmasked_500bp/44/44-1.png]

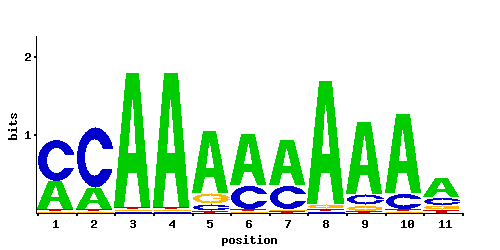

Supplement: Supplementary Data [file supp_btt248_Supplementary_Data.zip › Supplementary_Data/Results_Files/logos_non-repeatmasked_500bp/44/44-2.png]

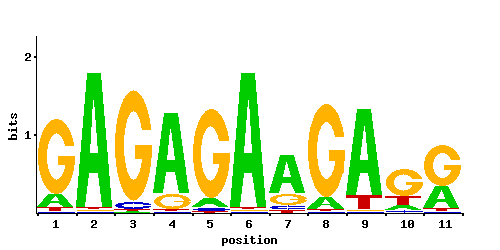

Supplement: Supplementary Data [file supp_btt248_Supplementary_Data.zip › Supplementary_Data/Results_Files/logos_non-repeatmasked_500bp/44/44-3.png]

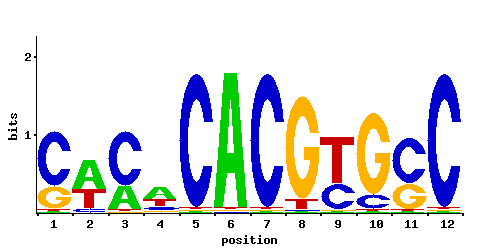

Supplement: Supplementary Data [file supp_btt248_Supplementary_Data.zip › Supplementary_Data/Results_Files/logos_non-repeatmasked_500bp/44/44-4.png]

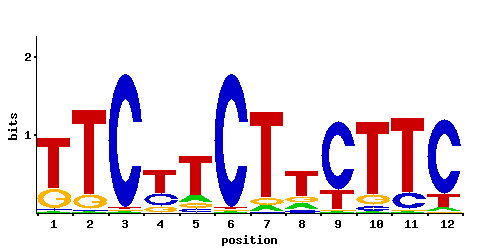

Supplement: Supplementary Data [file supp_btt248_Supplementary_Data.zip › Supplementary_Data/Results_Files/logos_non-repeatmasked_500bp/44/44-5.png]

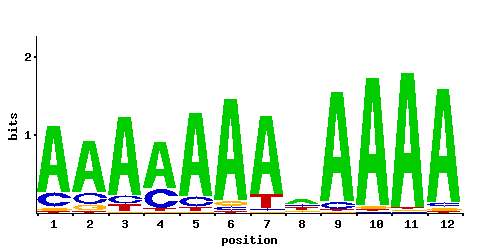

Supplement: Supplementary Data [file supp_btt248_Supplementary_Data.zip › Supplementary_Data/Results_Files/logos_non-repeatmasked_500bp/5/5-1.png]

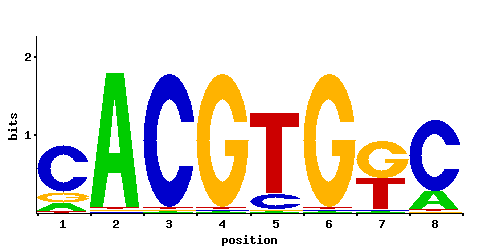

Supplement: Supplementary Data [file supp_btt248_Supplementary_Data.zip › Supplementary_Data/Results_Files/logos_non-repeatmasked_500bp/5/5-2.png]

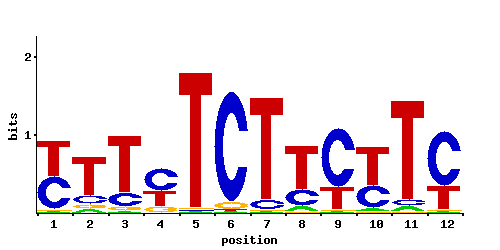

Supplement: Supplementary Data [file supp_btt248_Supplementary_Data.zip › Supplementary_Data/Results_Files/logos_non-repeatmasked_500bp/5/5-3.png]

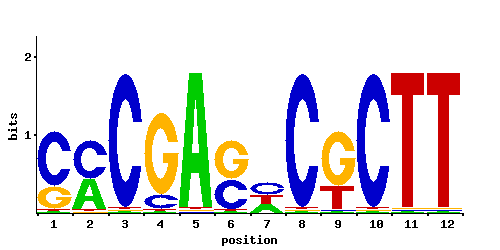

Supplement: Supplementary Data [file supp_btt248_Supplementary_Data.zip › Supplementary_Data/Results_Files/logos_non-repeatmasked_500bp/5/5-4.png]

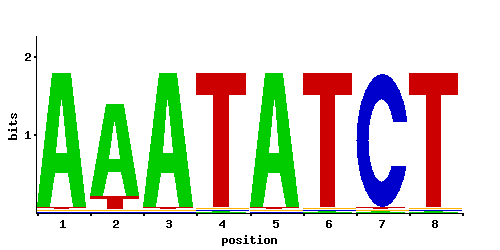

Supplement: Supplementary Data [file supp_btt248_Supplementary_Data.zip › Supplementary_Data/Results_Files/logos_non-repeatmasked_500bp/5/5-5.png]

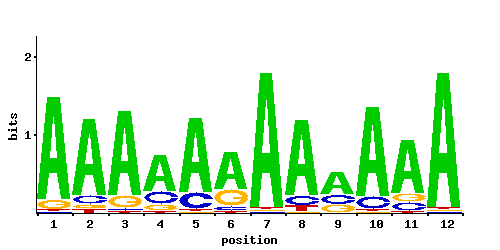

Supplement: Supplementary Data [file supp_btt248_Supplementary_Data.zip › Supplementary_Data/Results_Files/logos_non-repeatmasked_500bp/6/6-1.png]

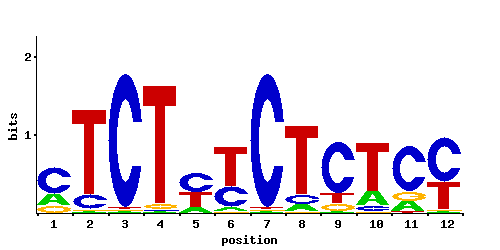

Supplement: Supplementary Data [file supp_btt248_Supplementary_Data.zip › Supplementary_Data/Results_Files/logos_non-repeatmasked_500bp/6/6-2.png]

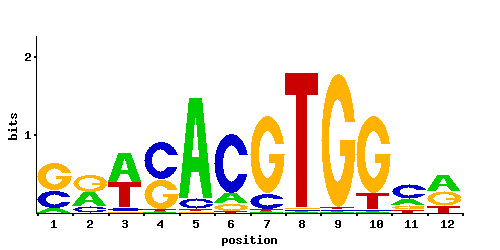

Supplement: Supplementary Data [file supp_btt248_Supplementary_Data.zip › Supplementary_Data/Results_Files/logos_non-repeatmasked_500bp/6/6-3.png]

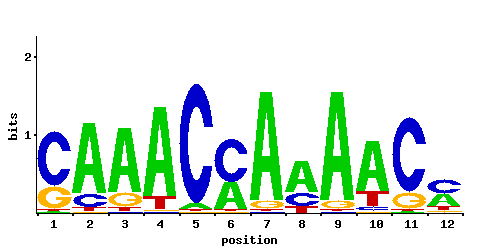

Supplement: Supplementary Data [file supp_btt248_Supplementary_Data.zip › Supplementary_Data/Results_Files/logos_non-repeatmasked_500bp/6/6-4.png]
