# Supplementary figures and images for: MEME-LaB: motif analysis in clusters (part 1 of 2)
Source: Bioinformatics. 2013 May 14;29(13):1696–7. doi: 10.1093/bioinformatics/btt248 (PMC3694638; doi:10.1093/bioinformatics/btt248)

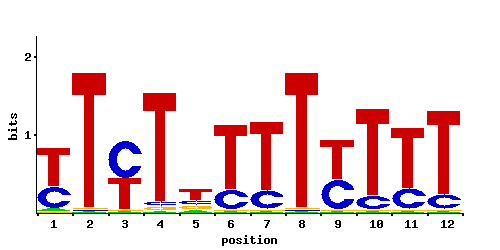

Supplement: Supplementary Data [file supp_btt248_Supplementary_Data.zip › Supplementary_Data/Results_Files/logos_non-repeatmasked_500bp/1/1-1.png]

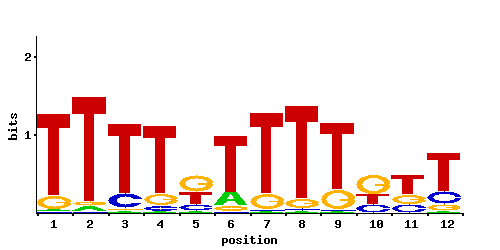

Supplement: Supplementary Data [file supp_btt248_Supplementary_Data.zip › Supplementary_Data/Results_Files/logos_non-repeatmasked_500bp/1/1-2.png]

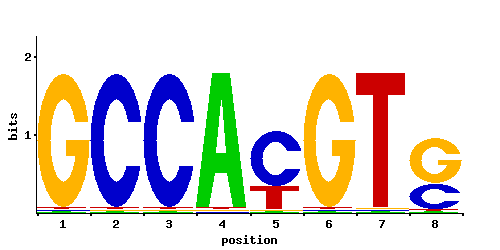

Supplement: Supplementary Data [file supp_btt248_Supplementary_Data.zip › Supplementary_Data/Results_Files/logos_non-repeatmasked_500bp/1/1-3.png]

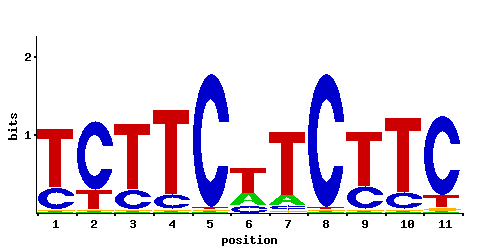

Supplement: Supplementary Data [file supp_btt248_Supplementary_Data.zip › Supplementary_Data/Results_Files/logos_non-repeatmasked_500bp/1/1-4.png]

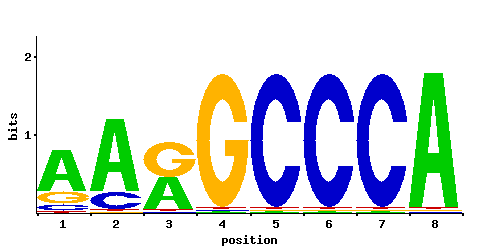

Supplement: Supplementary Data [file supp_btt248_Supplementary_Data.zip › Supplementary_Data/Results_Files/logos_non-repeatmasked_500bp/1/1-5.png]

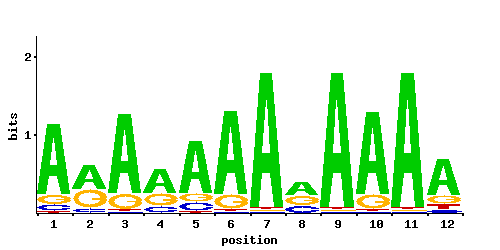

Supplement: Supplementary Data [file supp_btt248_Supplementary_Data.zip › Supplementary_Data/Results_Files/logos_non-repeatmasked_500bp/10/10-1.png]

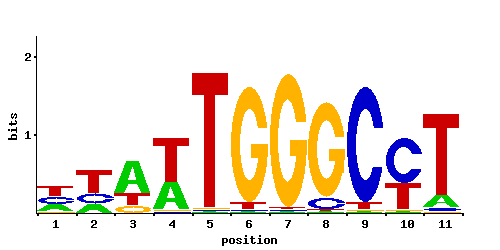

Supplement: Supplementary Data [file supp_btt248_Supplementary_Data.zip › Supplementary_Data/Results_Files/logos_non-repeatmasked_500bp/10/10-2.png]

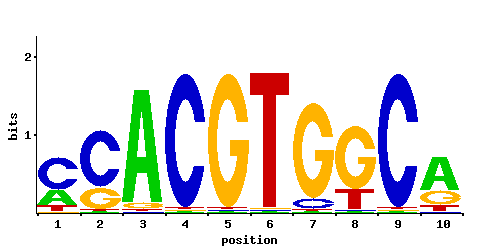

Supplement: Supplementary Data [file supp_btt248_Supplementary_Data.zip › Supplementary_Data/Results_Files/logos_non-repeatmasked_500bp/10/10-3.png]

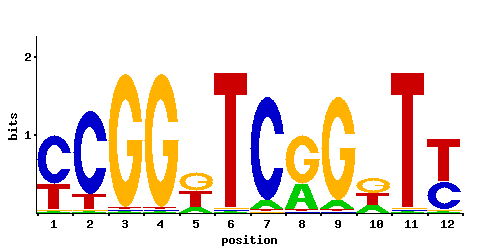

Supplement: Supplementary Data [file supp_btt248_Supplementary_Data.zip › Supplementary_Data/Results_Files/logos_non-repeatmasked_500bp/10/10-4.png]

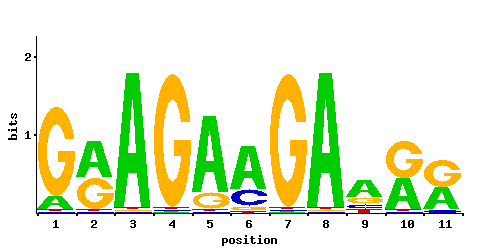

Supplement: Supplementary Data [file supp_btt248_Supplementary_Data.zip › Supplementary_Data/Results_Files/logos_non-repeatmasked_500bp/10/10-5.png]

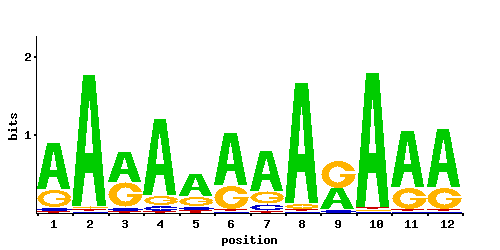

Supplement: Supplementary Data [file supp_btt248_Supplementary_Data.zip › Supplementary_Data/Results_Files/logos_non-repeatmasked_500bp/11/11-1.png]

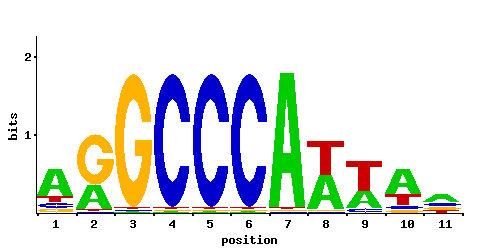

Supplement: Supplementary Data [file supp_btt248_Supplementary_Data.zip › Supplementary_Data/Results_Files/logos_non-repeatmasked_500bp/11/11-2.png]

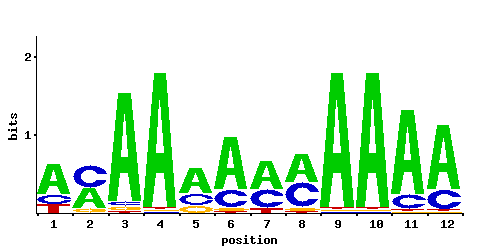

Supplement: Supplementary Data [file supp_btt248_Supplementary_Data.zip › Supplementary_Data/Results_Files/logos_non-repeatmasked_500bp/11/11-3.png]

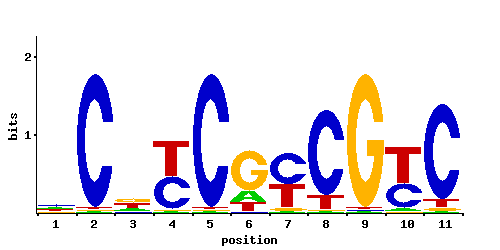

Supplement: Supplementary Data [file supp_btt248_Supplementary_Data.zip › Supplementary_Data/Results_Files/logos_non-repeatmasked_500bp/11/11-4.png]

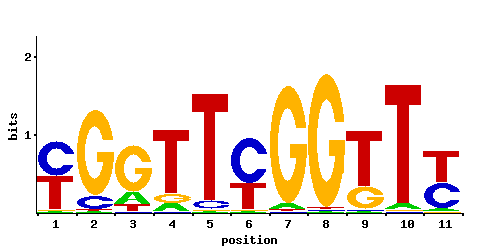

Supplement: Supplementary Data [file supp_btt248_Supplementary_Data.zip › Supplementary_Data/Results_Files/logos_non-repeatmasked_500bp/11/11-5.png]

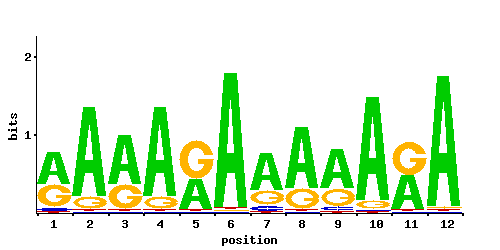

Supplement: Supplementary Data [file supp_btt248_Supplementary_Data.zip › Supplementary_Data/Results_Files/logos_non-repeatmasked_500bp/12/12-1.png]

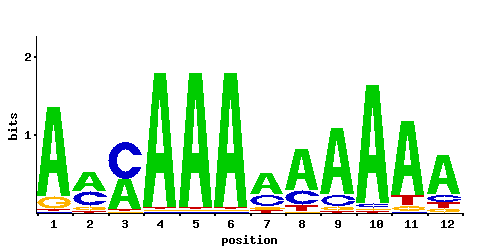

Supplement: Supplementary Data [file supp_btt248_Supplementary_Data.zip › Supplementary_Data/Results_Files/logos_non-repeatmasked_500bp/12/12-2.png]

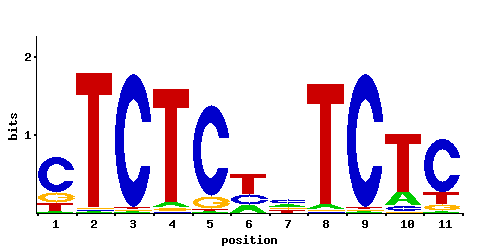

Supplement: Supplementary Data [file supp_btt248_Supplementary_Data.zip › Supplementary_Data/Results_Files/logos_non-repeatmasked_500bp/12/12-3.png]

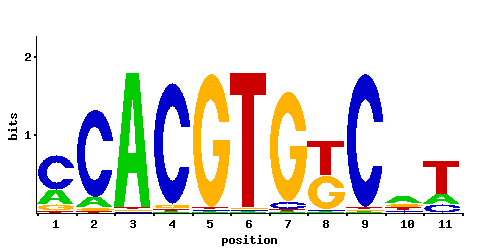

Supplement: Supplementary Data [file supp_btt248_Supplementary_Data.zip › Supplementary_Data/Results_Files/logos_non-repeatmasked_500bp/12/12-4.png]

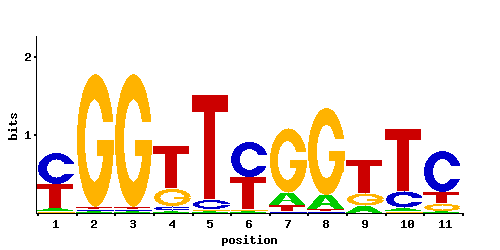

Supplement: Supplementary Data [file supp_btt248_Supplementary_Data.zip › Supplementary_Data/Results_Files/logos_non-repeatmasked_500bp/12/12-5.png]

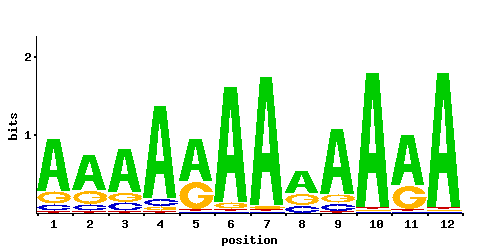

Supplement: Supplementary Data [file supp_btt248_Supplementary_Data.zip › Supplementary_Data/Results_Files/logos_non-repeatmasked_500bp/13/13-1.png]

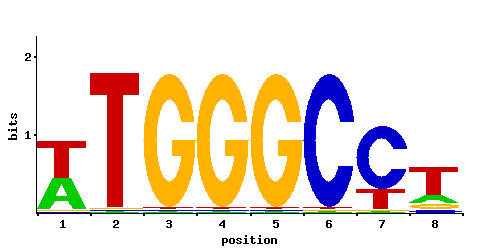

Supplement: Supplementary Data [file supp_btt248_Supplementary_Data.zip › Supplementary_Data/Results_Files/logos_non-repeatmasked_500bp/13/13-2.png]

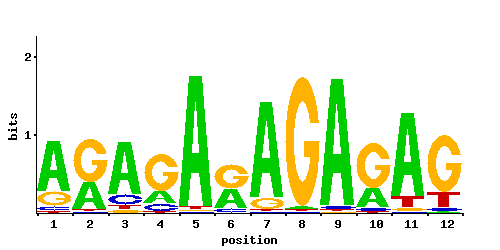

Supplement: Supplementary Data [file supp_btt248_Supplementary_Data.zip › Supplementary_Data/Results_Files/logos_non-repeatmasked_500bp/13/13-3.png]

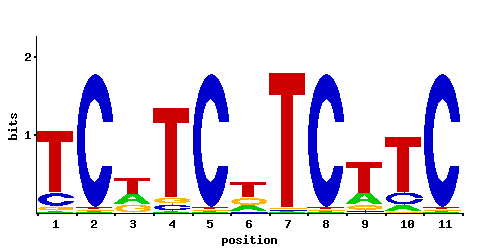

Supplement: Supplementary Data [file supp_btt248_Supplementary_Data.zip › Supplementary_Data/Results_Files/logos_non-repeatmasked_500bp/13/13-4.png]

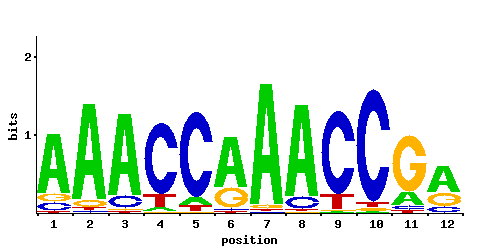

Supplement: Supplementary Data [file supp_btt248_Supplementary_Data.zip › Supplementary_Data/Results_Files/logos_non-repeatmasked_500bp/13/13-5.png]

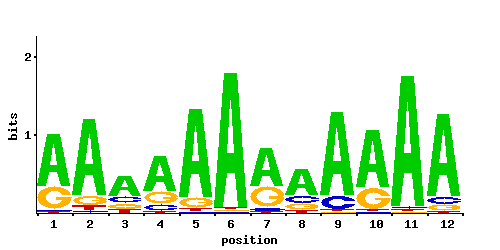

Supplement: Supplementary Data [file supp_btt248_Supplementary_Data.zip › Supplementary_Data/Results_Files/logos_non-repeatmasked_500bp/14/14-1.png]

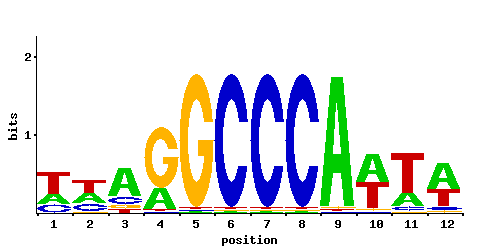

Supplement: Supplementary Data [file supp_btt248_Supplementary_Data.zip › Supplementary_Data/Results_Files/logos_non-repeatmasked_500bp/14/14-2.png]

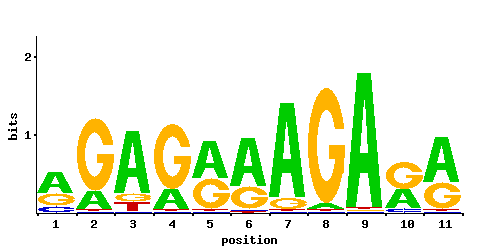

Supplement: Supplementary Data [file supp_btt248_Supplementary_Data.zip › Supplementary_Data/Results_Files/logos_non-repeatmasked_500bp/14/14-3.png]

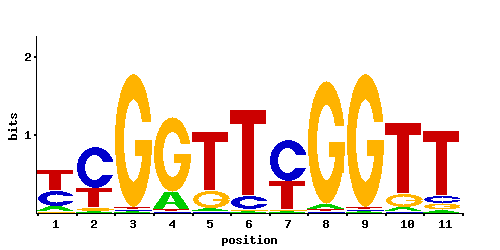

Supplement: Supplementary Data [file supp_btt248_Supplementary_Data.zip › Supplementary_Data/Results_Files/logos_non-repeatmasked_500bp/14/14-4.png]

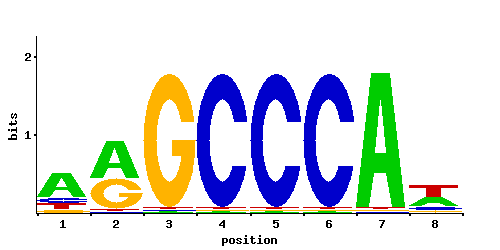

Supplement: Supplementary Data [file supp_btt248_Supplementary_Data.zip › Supplementary_Data/Results_Files/logos_non-repeatmasked_500bp/14/14-5.png]

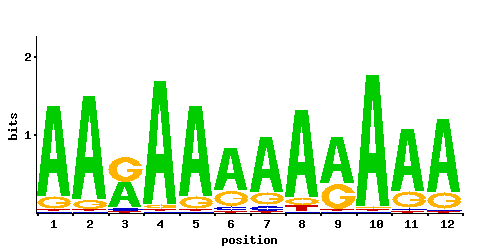

Supplement: Supplementary Data [file supp_btt248_Supplementary_Data.zip › Supplementary_Data/Results_Files/logos_non-repeatmasked_500bp/15/15-1.png]

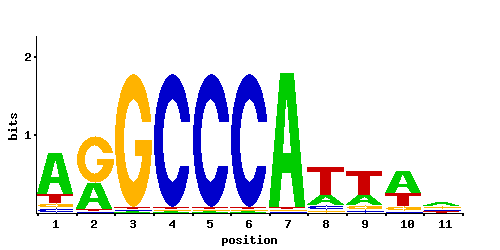

Supplement: Supplementary Data [file supp_btt248_Supplementary_Data.zip › Supplementary_Data/Results_Files/logos_non-repeatmasked_500bp/15/15-2.png]

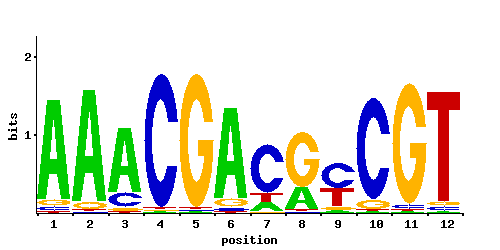

Supplement: Supplementary Data [file supp_btt248_Supplementary_Data.zip › Supplementary_Data/Results_Files/logos_non-repeatmasked_500bp/15/15-3.png]

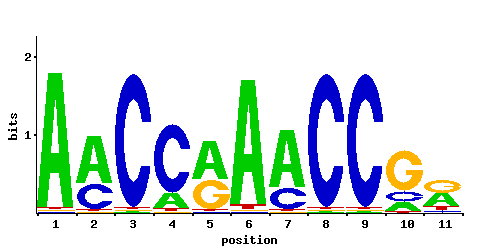

Supplement: Supplementary Data [file supp_btt248_Supplementary_Data.zip › Supplementary_Data/Results_Files/logos_non-repeatmasked_500bp/15/15-4.png]

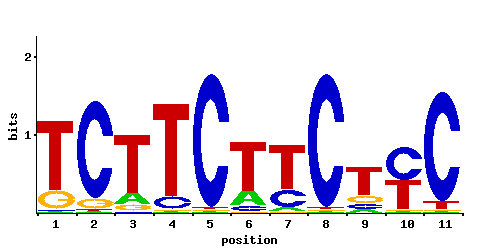

Supplement: Supplementary Data [file supp_btt248_Supplementary_Data.zip › Supplementary_Data/Results_Files/logos_non-repeatmasked_500bp/15/15-5.png]

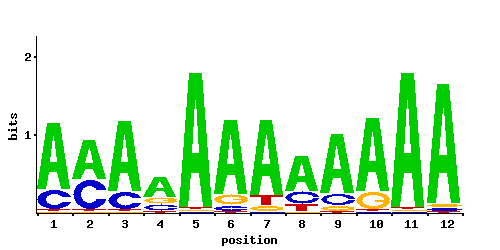

Supplement: Supplementary Data [file supp_btt248_Supplementary_Data.zip › Supplementary_Data/Results_Files/logos_non-repeatmasked_500bp/16/16-1.png]

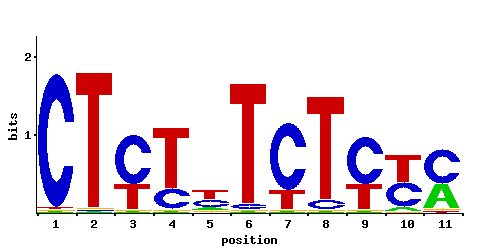

Supplement: Supplementary Data [file supp_btt248_Supplementary_Data.zip › Supplementary_Data/Results_Files/logos_non-repeatmasked_500bp/16/16-2.png]

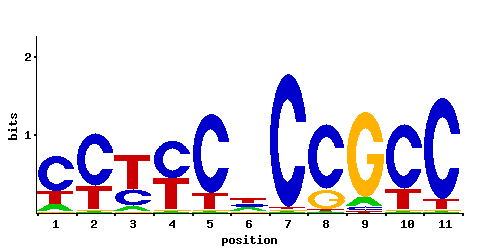

Supplement: Supplementary Data [file supp_btt248_Supplementary_Data.zip › Supplementary_Data/Results_Files/logos_non-repeatmasked_500bp/16/16-3.png]

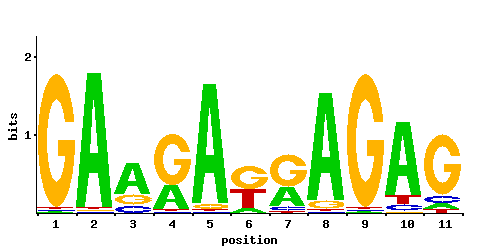

Supplement: Supplementary Data [file supp_btt248_Supplementary_Data.zip › Supplementary_Data/Results_Files/logos_non-repeatmasked_500bp/16/16-4.png]

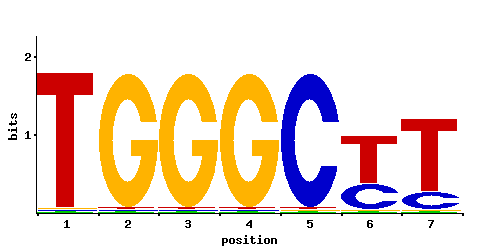

Supplement: Supplementary Data [file supp_btt248_Supplementary_Data.zip › Supplementary_Data/Results_Files/logos_non-repeatmasked_500bp/16/16-5.png]

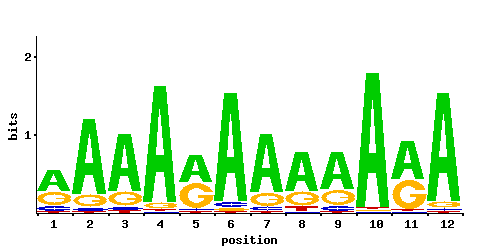

Supplement: Supplementary Data [file supp_btt248_Supplementary_Data.zip › Supplementary_Data/Results_Files/logos_non-repeatmasked_500bp/18/18-1.png]

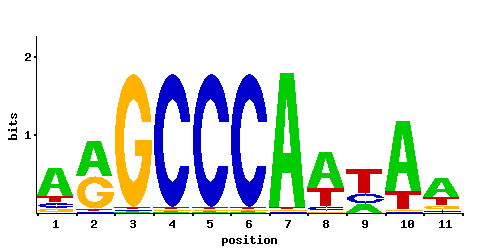

Supplement: Supplementary Data [file supp_btt248_Supplementary_Data.zip › Supplementary_Data/Results_Files/logos_non-repeatmasked_500bp/18/18-2.png]

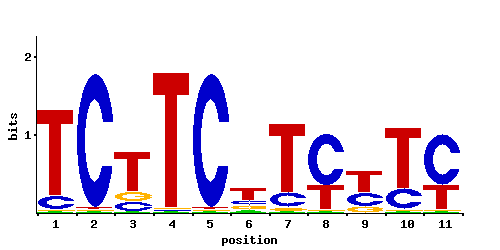

Supplement: Supplementary Data [file supp_btt248_Supplementary_Data.zip › Supplementary_Data/Results_Files/logos_non-repeatmasked_500bp/18/18-3.png]

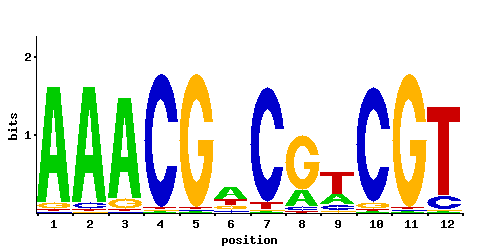

Supplement: Supplementary Data [file supp_btt248_Supplementary_Data.zip › Supplementary_Data/Results_Files/logos_non-repeatmasked_500bp/18/18-4.png]

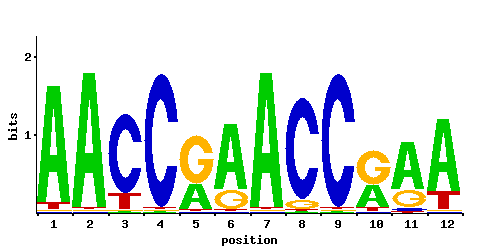

Supplement: Supplementary Data [file supp_btt248_Supplementary_Data.zip › Supplementary_Data/Results_Files/logos_non-repeatmasked_500bp/18/18-5.png]

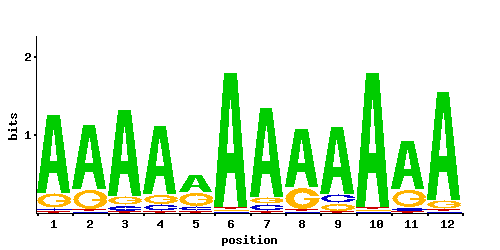

Supplement: Supplementary Data [file supp_btt248_Supplementary_Data.zip › Supplementary_Data/Results_Files/logos_non-repeatmasked_500bp/19/19-1.png]

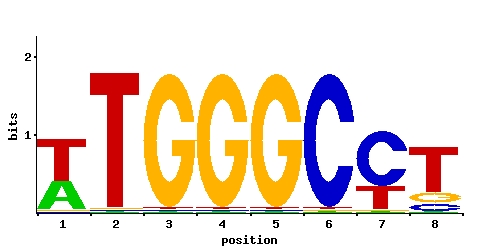

Supplement: Supplementary Data [file supp_btt248_Supplementary_Data.zip › Supplementary_Data/Results_Files/logos_non-repeatmasked_500bp/19/19-2.png]

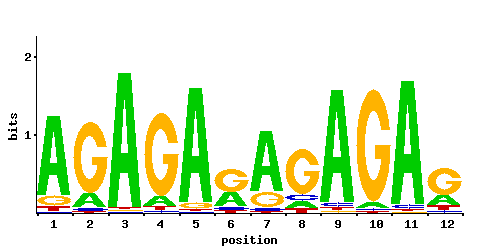

Supplement: Supplementary Data [file supp_btt248_Supplementary_Data.zip › Supplementary_Data/Results_Files/logos_non-repeatmasked_500bp/19/19-3.png]

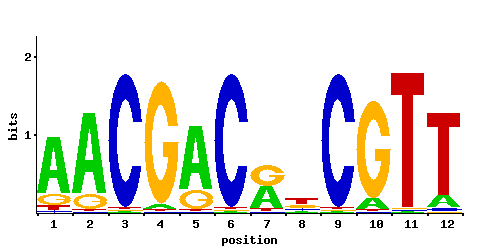

Supplement: Supplementary Data [file supp_btt248_Supplementary_Data.zip › Supplementary_Data/Results_Files/logos_non-repeatmasked_500bp/19/19-4.png]

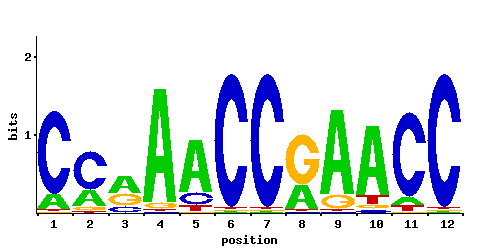

Supplement: Supplementary Data [file supp_btt248_Supplementary_Data.zip › Supplementary_Data/Results_Files/logos_non-repeatmasked_500bp/19/19-5.png]

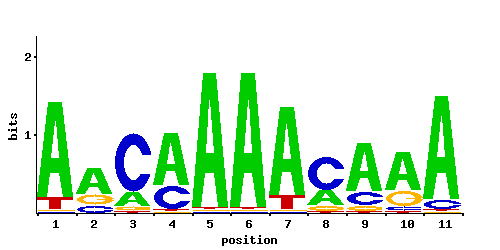

Supplement: Supplementary Data [file supp_btt248_Supplementary_Data.zip › Supplementary_Data/Results_Files/logos_non-repeatmasked_500bp/2/2-1.png]

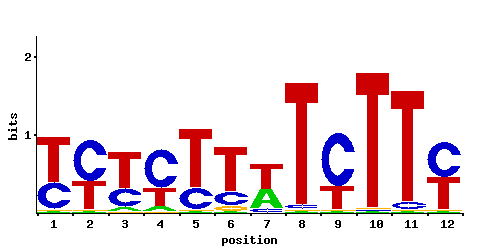

Supplement: Supplementary Data [file supp_btt248_Supplementary_Data.zip › Supplementary_Data/Results_Files/logos_non-repeatmasked_500bp/2/2-2.png]

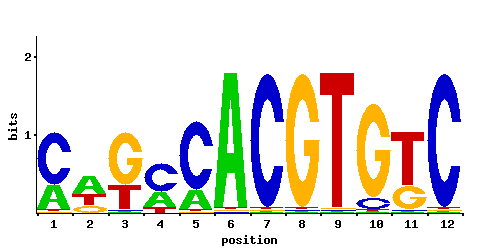

Supplement: Supplementary Data [file supp_btt248_Supplementary_Data.zip › Supplementary_Data/Results_Files/logos_non-repeatmasked_500bp/2/2-3.png]

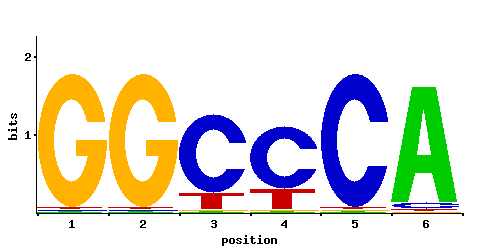

Supplement: Supplementary Data [file supp_btt248_Supplementary_Data.zip › Supplementary_Data/Results_Files/logos_non-repeatmasked_500bp/2/2-4.png]

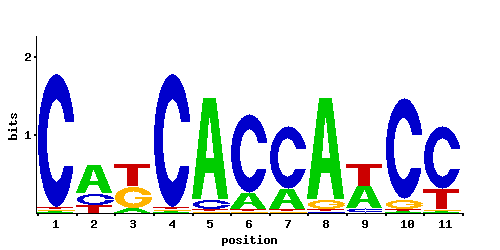

Supplement: Supplementary Data [file supp_btt248_Supplementary_Data.zip › Supplementary_Data/Results_Files/logos_non-repeatmasked_500bp/2/2-5.png]

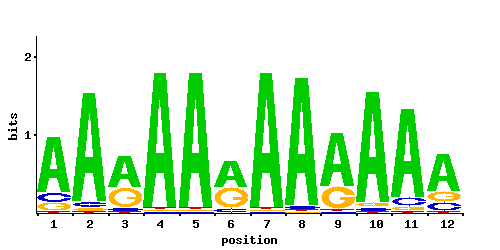

Supplement: Supplementary Data [file supp_btt248_Supplementary_Data.zip › Supplementary_Data/Results_Files/logos_non-repeatmasked_500bp/20/20-1.png]

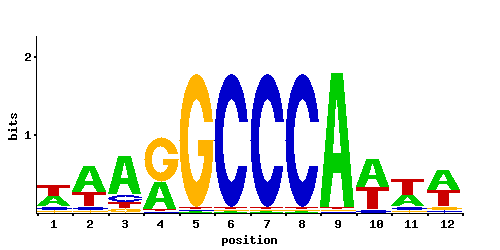

Supplement: Supplementary Data [file supp_btt248_Supplementary_Data.zip › Supplementary_Data/Results_Files/logos_non-repeatmasked_500bp/20/20-2.png]

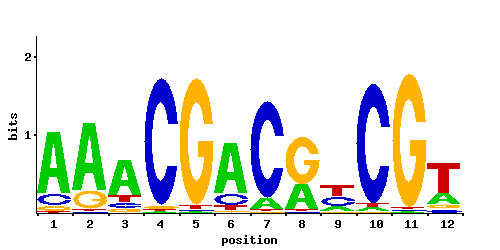

Supplement: Supplementary Data [file supp_btt248_Supplementary_Data.zip › Supplementary_Data/Results_Files/logos_non-repeatmasked_500bp/20/20-3.png]

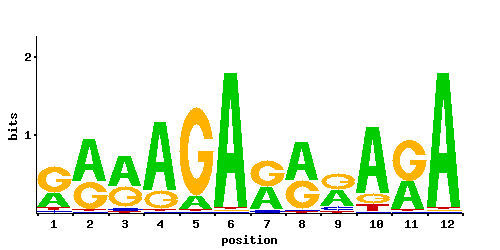

Supplement: Supplementary Data [file supp_btt248_Supplementary_Data.zip › Supplementary_Data/Results_Files/logos_non-repeatmasked_500bp/20/20-4.png]

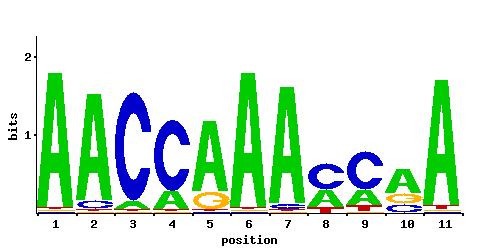

Supplement: Supplementary Data [file supp_btt248_Supplementary_Data.zip › Supplementary_Data/Results_Files/logos_non-repeatmasked_500bp/20/20-5.png]

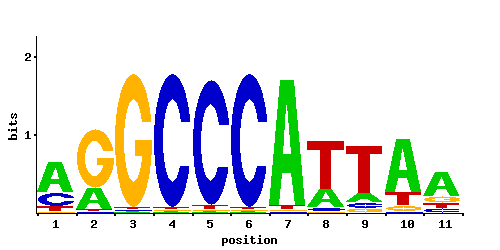

Supplement: Supplementary Data [file supp_btt248_Supplementary_Data.zip › Supplementary_Data/Results_Files/logos_non-repeatmasked_500bp/21/21-1.png]

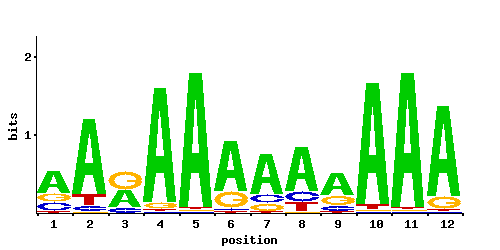

Supplement: Supplementary Data [file supp_btt248_Supplementary_Data.zip › Supplementary_Data/Results_Files/logos_non-repeatmasked_500bp/21/21-2.png]

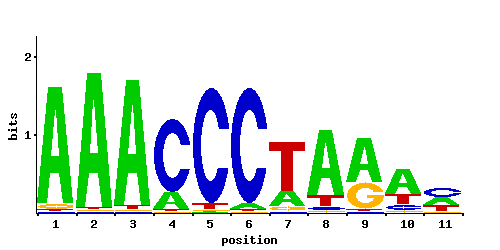

Supplement: Supplementary Data [file supp_btt248_Supplementary_Data.zip › Supplementary_Data/Results_Files/logos_non-repeatmasked_500bp/21/21-3.png]

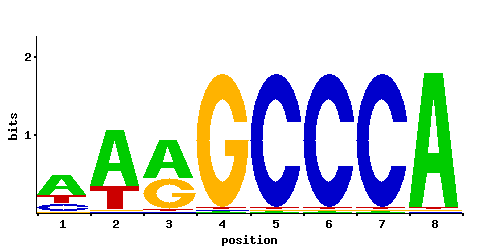

Supplement: Supplementary Data [file supp_btt248_Supplementary_Data.zip › Supplementary_Data/Results_Files/logos_non-repeatmasked_500bp/21/21-4.png]

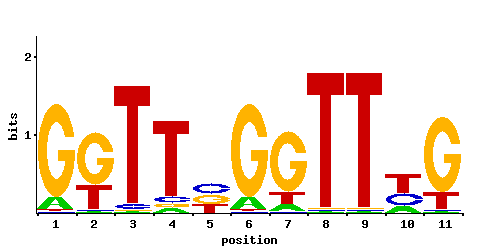

Supplement: Supplementary Data [file supp_btt248_Supplementary_Data.zip › Supplementary_Data/Results_Files/logos_non-repeatmasked_500bp/21/21-5.png]

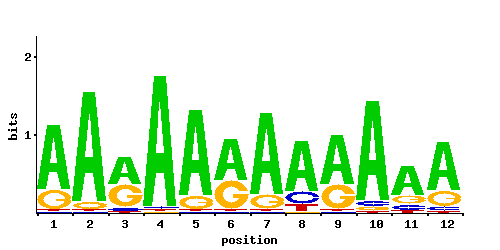

Supplement: Supplementary Data [file supp_btt248_Supplementary_Data.zip › Supplementary_Data/Results_Files/logos_non-repeatmasked_500bp/22/22-1.png]

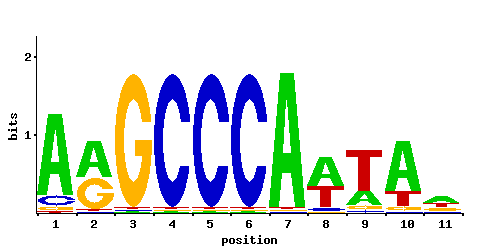

Supplement: Supplementary Data [file supp_btt248_Supplementary_Data.zip › Supplementary_Data/Results_Files/logos_non-repeatmasked_500bp/22/22-2.png]

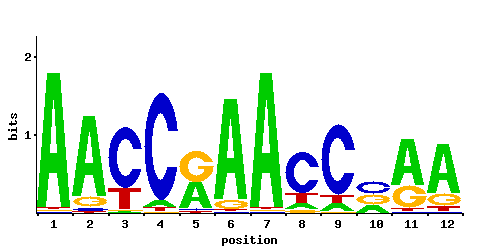

Supplement: Supplementary Data [file supp_btt248_Supplementary_Data.zip › Supplementary_Data/Results_Files/logos_non-repeatmasked_500bp/22/22-3.png]

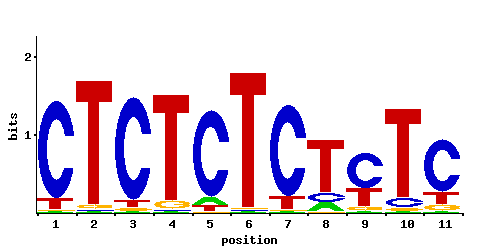

Supplement: Supplementary Data [file supp_btt248_Supplementary_Data.zip › Supplementary_Data/Results_Files/logos_non-repeatmasked_500bp/22/22-4.png]

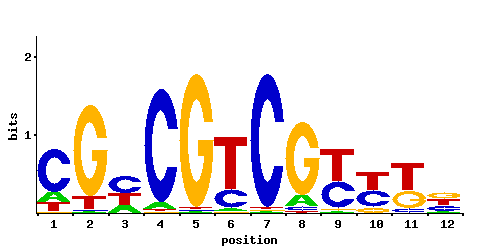

Supplement: Supplementary Data [file supp_btt248_Supplementary_Data.zip › Supplementary_Data/Results_Files/logos_non-repeatmasked_500bp/22/22-5.png]

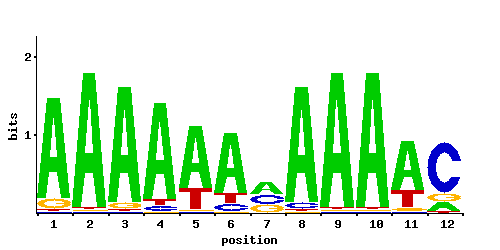

Supplement: Supplementary Data [file supp_btt248_Supplementary_Data.zip › Supplementary_Data/Results_Files/logos_non-repeatmasked_500bp/23/23-1.png]

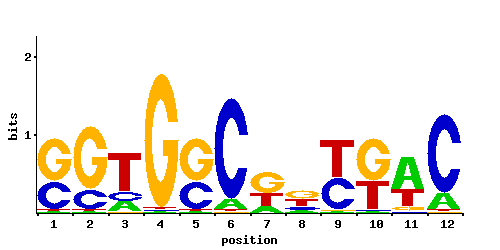

Supplement: Supplementary Data [file supp_btt248_Supplementary_Data.zip › Supplementary_Data/Results_Files/logos_non-repeatmasked_500bp/23/23-2.png]

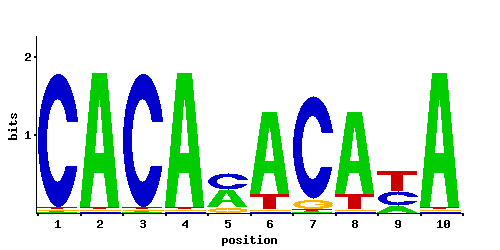

Supplement: Supplementary Data [file supp_btt248_Supplementary_Data.zip › Supplementary_Data/Results_Files/logos_non-repeatmasked_500bp/23/23-3.png]

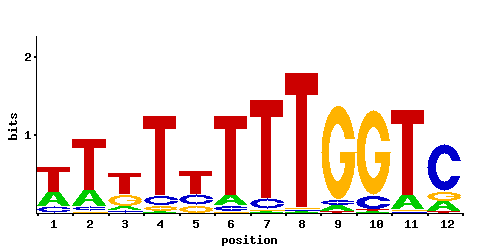

Supplement: Supplementary Data [file supp_btt248_Supplementary_Data.zip › Supplementary_Data/Results_Files/logos_non-repeatmasked_500bp/23/23-4.png]

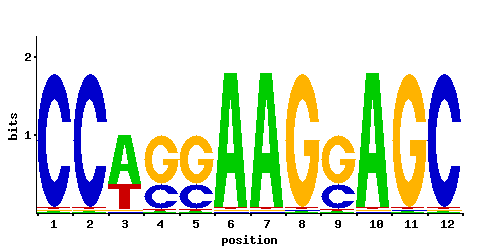

Supplement: Supplementary Data [file supp_btt248_Supplementary_Data.zip › Supplementary_Data/Results_Files/logos_non-repeatmasked_500bp/23/23-5.png]

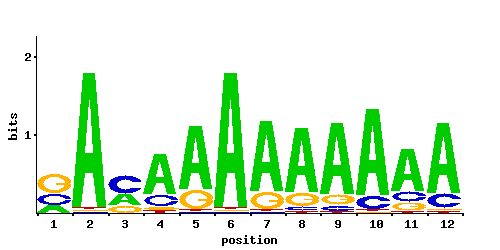

Supplement: Supplementary Data [file supp_btt248_Supplementary_Data.zip › Supplementary_Data/Results_Files/logos_non-repeatmasked_500bp/24/24-1.png]

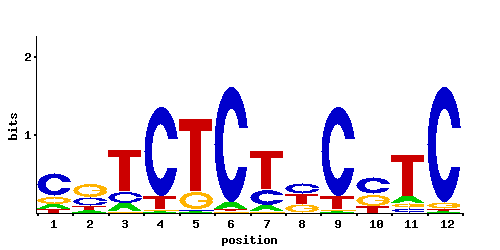

Supplement: Supplementary Data [file supp_btt248_Supplementary_Data.zip › Supplementary_Data/Results_Files/logos_non-repeatmasked_500bp/24/24-2.png]

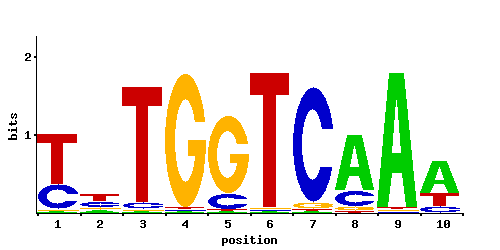

Supplement: Supplementary Data [file supp_btt248_Supplementary_Data.zip › Supplementary_Data/Results_Files/logos_non-repeatmasked_500bp/24/24-3.png]

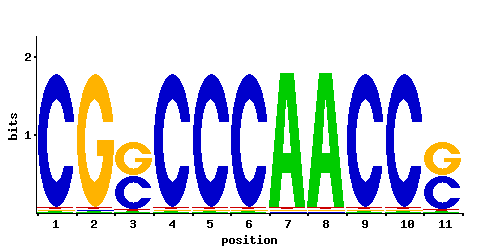

Supplement: Supplementary Data [file supp_btt248_Supplementary_Data.zip › Supplementary_Data/Results_Files/logos_non-repeatmasked_500bp/24/24-4.png]

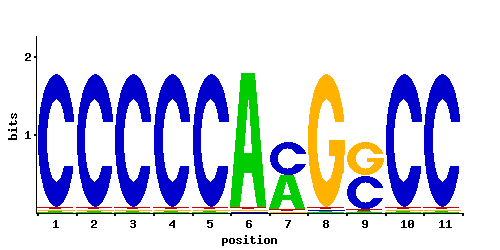

Supplement: Supplementary Data [file supp_btt248_Supplementary_Data.zip › Supplementary_Data/Results_Files/logos_non-repeatmasked_500bp/24/24-5.png]

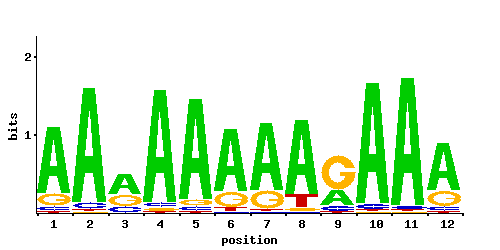

Supplement: Supplementary Data [file supp_btt248_Supplementary_Data.zip › Supplementary_Data/Results_Files/logos_non-repeatmasked_500bp/25/25-1.png]

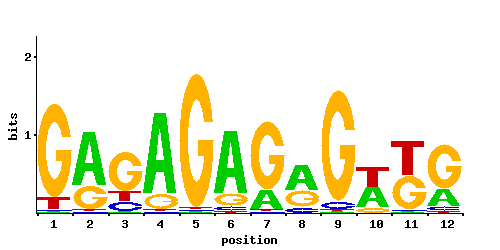

Supplement: Supplementary Data [file supp_btt248_Supplementary_Data.zip › Supplementary_Data/Results_Files/logos_non-repeatmasked_500bp/25/25-2.png]

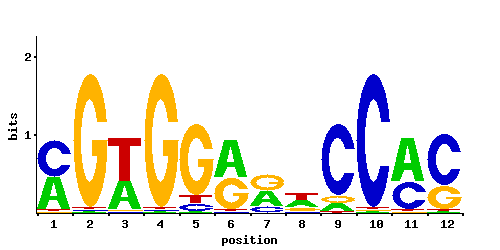

Supplement: Supplementary Data [file supp_btt248_Supplementary_Data.zip › Supplementary_Data/Results_Files/logos_non-repeatmasked_500bp/25/25-3.png]

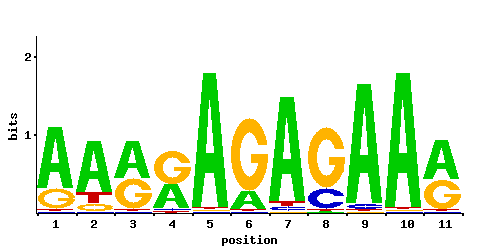

Supplement: Supplementary Data [file supp_btt248_Supplementary_Data.zip › Supplementary_Data/Results_Files/logos_non-repeatmasked_500bp/25/25-4.png]

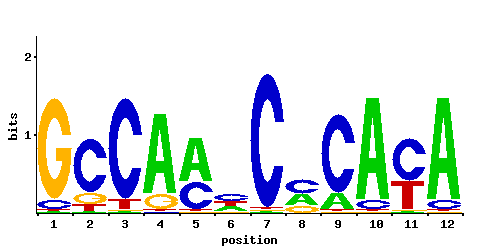

Supplement: Supplementary Data [file supp_btt248_Supplementary_Data.zip › Supplementary_Data/Results_Files/logos_non-repeatmasked_500bp/25/25-5.png]

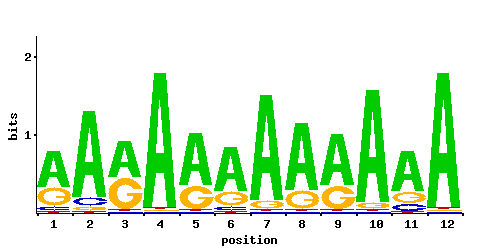

Supplement: Supplementary Data [file supp_btt248_Supplementary_Data.zip › Supplementary_Data/Results_Files/logos_non-repeatmasked_500bp/26/26-1.png]

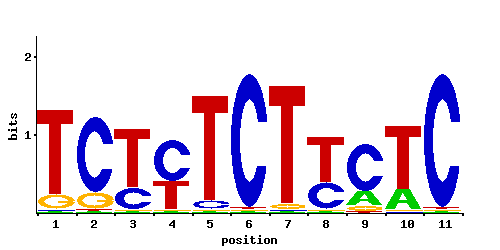

Supplement: Supplementary Data [file supp_btt248_Supplementary_Data.zip › Supplementary_Data/Results_Files/logos_non-repeatmasked_500bp/26/26-2.png]

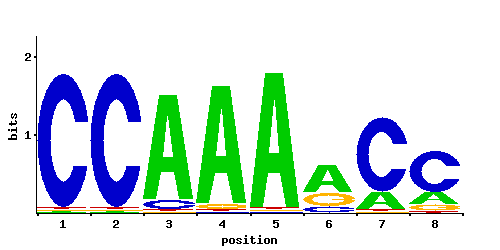

Supplement: Supplementary Data [file supp_btt248_Supplementary_Data.zip › Supplementary_Data/Results_Files/logos_non-repeatmasked_500bp/26/26-3.png]

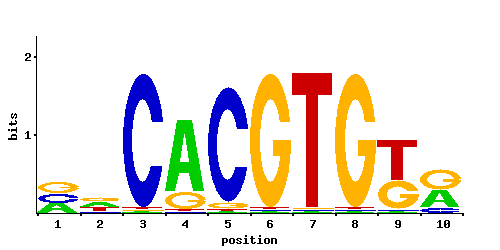

Supplement: Supplementary Data [file supp_btt248_Supplementary_Data.zip › Supplementary_Data/Results_Files/logos_non-repeatmasked_500bp/26/26-4.png]

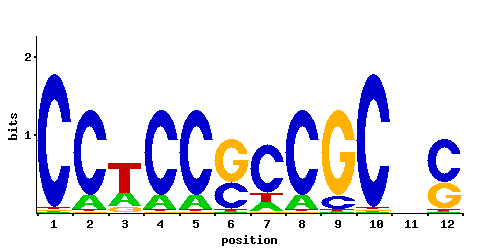

Supplement: Supplementary Data [file supp_btt248_Supplementary_Data.zip › Supplementary_Data/Results_Files/logos_non-repeatmasked_500bp/26/26-5.png]

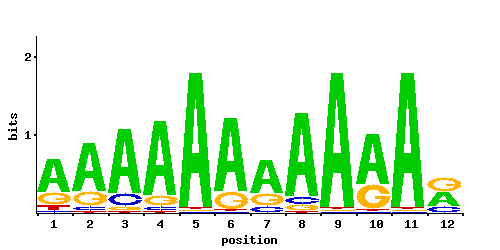

Supplement: Supplementary Data [file supp_btt248_Supplementary_Data.zip › Supplementary_Data/Results_Files/logos_non-repeatmasked_500bp/27/27-1.png]

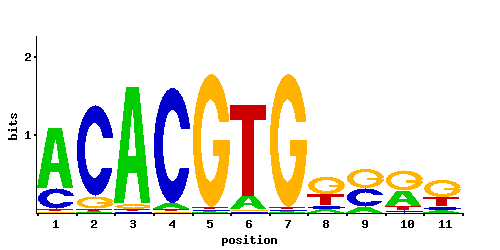

Supplement: Supplementary Data [file supp_btt248_Supplementary_Data.zip › Supplementary_Data/Results_Files/logos_non-repeatmasked_500bp/27/27-2.png]

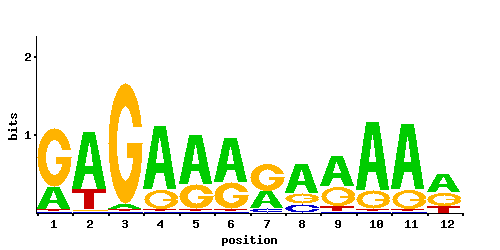

Supplement: Supplementary Data [file supp_btt248_Supplementary_Data.zip › Supplementary_Data/Results_Files/logos_non-repeatmasked_500bp/27/27-3.png]

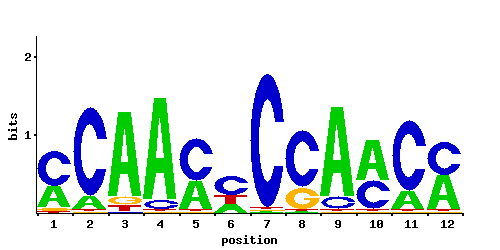

Supplement: Supplementary Data [file supp_btt248_Supplementary_Data.zip › Supplementary_Data/Results_Files/logos_non-repeatmasked_500bp/27/27-4.png]

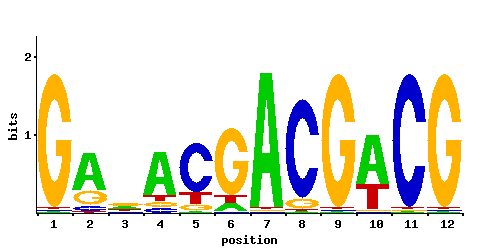

Supplement: Supplementary Data [file supp_btt248_Supplementary_Data.zip › Supplementary_Data/Results_Files/logos_non-repeatmasked_500bp/27/27-5.png]

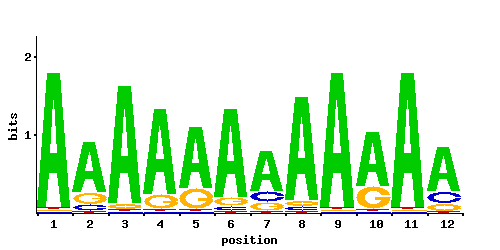

Supplement: Supplementary Data [file supp_btt248_Supplementary_Data.zip › Supplementary_Data/Results_Files/logos_non-repeatmasked_500bp/28/28-1.png]

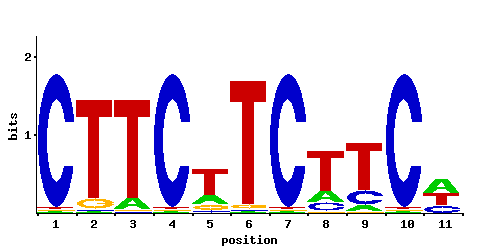

Supplement: Supplementary Data [file supp_btt248_Supplementary_Data.zip › Supplementary_Data/Results_Files/logos_non-repeatmasked_500bp/28/28-2.png]

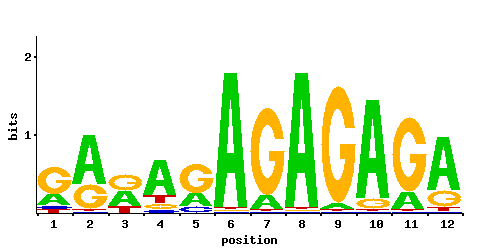

Supplement: Supplementary Data [file supp_btt248_Supplementary_Data.zip › Supplementary_Data/Results_Files/logos_non-repeatmasked_500bp/28/28-3.png]

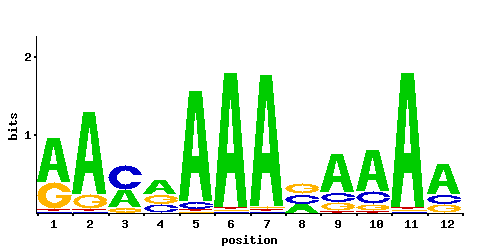

Supplement: Supplementary Data [file supp_btt248_Supplementary_Data.zip › Supplementary_Data/Results_Files/logos_non-repeatmasked_500bp/28/28-4.png]

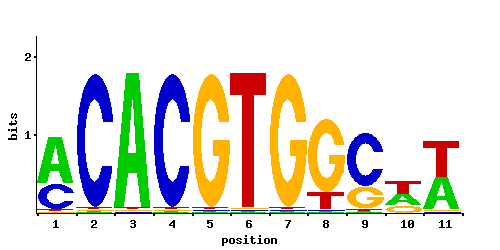

Supplement: Supplementary Data [file supp_btt248_Supplementary_Data.zip › Supplementary_Data/Results_Files/logos_non-repeatmasked_500bp/28/28-5.png]
